# Graphene Field-Effect Transistor Process and Analysis Optimization in 200 mm Pilot-Line Environment

**DOI:** 10.1021/acsnano.6c03282

**Published:** 2026-09-09

**Authors:** Anton Murros, Miika Soikkeli, Anni Virta, Arantxa Maestre, Leire Morillo, Alba Centeno, Amaia Zurutuza, Olli-Pekka Kilpi

**Affiliations:** † 3259 VTT Technical Research Centre of Finland Ltd, P.O. Box 1000, Espoo FI-02044 VTT, Finland; ‡ 383835 Graphenea S.A., Paseo Mikeletegi 83, San Sebastian 20009, Spain; § ASM International, Kapeldreef 75, Leuven 3001, Belgium

**Keywords:** graphene, field-effect transistor, wafer-scale, processing, analysis, mobility, contact resistance

## Abstract

The maturity of the chemical vapor deposition graphene-based device processing has increased from chip-level demonstrations to wafer-scale fabrication in the past few years. Due to this, wafer-scale electrical characterization and analysis of the fabricated devices have become increasingly important to enable extraction of multiple parameters with a minimal number of measurements for the quality-control purposes critical for industrial uptake of 2D material-based devices. As a crucial step, we demonstrate optimization of complementary metal-oxide semiconductor (CMOS) back-end-of-line (BEOL)-compatible graphene field-effect transistor (GFET) fabrication and analysis, including the gate stack, bottom contact, graphene patterning, and encapsulation process steps. The analysis methods include atomic force microscopy, scanning electron microscopy, and, most importantly, electrical characterization. The electrical characterization focuses on comparing different test structures and extraction methods for mobility, contact resistance, transfer curve hysteresis, and doping parameters. The comparison shows that the selected measurement test structures and analysis methods can have a large impact on the extracted values and should thus be considered when comparing data sets from different sources. The analysis shows that the optimized process offers a high device yield of 97% with good doping uniformity, contact resistance, and mobility as well as low transfer-curve hysteresis values on 200 mm wafers.

## Graphical abstract

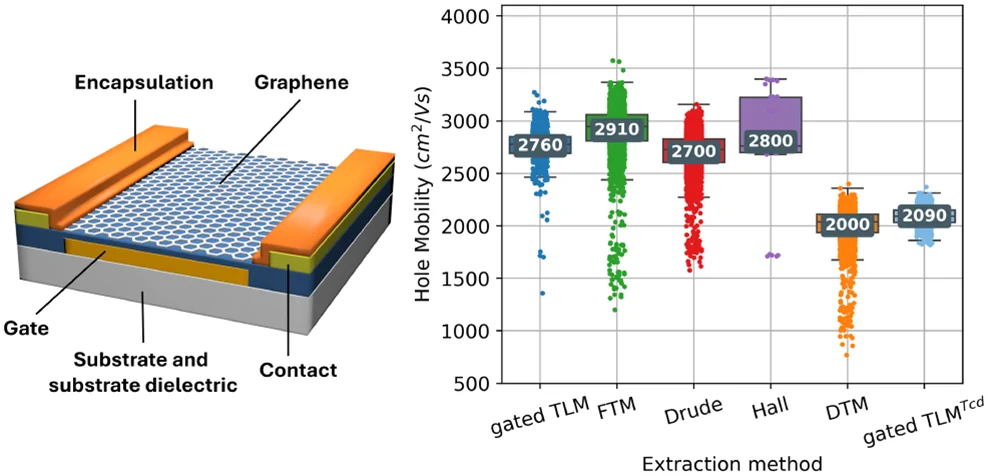

## Introduction

Graphene, first isolated in 2004 using mechanical exfoliation, marked the beginning of a new era in two-dimensional material research due to its exceptional electrical and mechanical properties.^1^ Currently, after over 20 years of research, the maturity of graphene-based technologies is transitioning from chip-level fabrication and analysis of single devices to wafer-scale fabrication and statistical analysis of numerous devices within a wafer and between different wafers. Wafer-scale chemical vapor deposition (CVD) graphene-based device fabrication has already been demonstrated on 200 mm and 300 mm wafer platforms for sensing and photonics application areas thanks to the advancements in the growth, transfer, and processing of the 2D materials made in recent years.^2–9^ These advancements are critical for the industrial uptake of the 2D materials in clearing the barriers for integration into semiconductor fabrication lines.^10^

It is critical to ensure that the CVD graphene-based sensor fabrication processes are compatible with CMOS back-end-of-line (BEOL), as this can bring the applications to a higher maturity level, for example, for broadband image detectors,^11^ gas sensing,^12^ and biosensing.^4^ The relatively advanced technological level achieved in these studies shows significant progress in the monolithic integration of 2D material sensors into advanced CMOS systems. The possibilities and challenges of graphene integration into CMOS back-end-of-line (BEOL) have been discussed by Neumaier et al.^10^ However, many of the presented integration flows only offer limited functionality of the devices in CMOS BEOL integration for sensing applications due to the lack of gates,^11^ global Si back gates,^13^ and liquid gates^4^ instead of local back gates. The local back gates offer clear benefits for quality control and sensitivity tuning of the devices.^2^ These are especially beneficial for gas sensors^14^ and photodetectors,^2^ while biosensors^4,15^ typically favor the usage of liquid gates instead of local back gates in actual biosensing scenarios but can also gain increased sensitivity with dual-gate^16,17^ sensing configurations.

Large-scale adaptation of GFETs requires quality-control protocols with detailed extraction of multiple parameters for detailed quality control of the fabricated GFETs;^18,19^ however, measurements and analyses of wafer-scale GFETs are often conducted to extract only a single or a few electrical parameters such as mobility,^13^ contact resistance,^20–22^ doping,^13^ and transfer-curve hysteresis,^23^ which might be adequate for a specific application but only provide limited information on the quality of the devices.^17,18^ The measurement conditions in which the device characterization is performed should also be carefully considered since the ambient measurement conditions heavily affect the obtained parameters, and thus, the device parameters should be obtained either in vacuum^24,25^ or with passivated devices.^13^

Electrical characterization of graphene-based devices is typically done by using, for example, the transfer length method (TLM) with and without gating,^26,27^ Hall bar,^28^ van der Pauw,^29,30^ cross-bridge Kelvin (CBK),^31,32^ contact-end-resistance (CER),^25,32–34^ GFET,^13^ and capacitor^35^ test structures. These methods are targeted to extract material and device parameters, such as contact resistance, doping, and mobility. Different structures have pros and cons that affect the reliability of these methods. Gated TLM structures offer the possibility to normalize the transfer curves with respect to the Dirac peak position, leading to more accurate extraction of parameters at specific carrier concentrations. Hall bar and van der Pauw structures can be utilized to measure sheet resistance of the graphene devices directly with 4-point measurement configuration for mobility extraction.^28–30^ Mobility calculation from the extracted or measured sheet resistance values can then be done, for example, by using transconductance or Drude methods. As a complementary contact resistance extraction method for graphene devices, CBK and CER structures have been utilized.^34,36^ Individual GFETs can be directly analyzed by using the direct transconductance method (DTM) for mobility or by a fitting method (FTM)^37^ for mobility and contact resistance values.^26^ For accurate estimation of the mobility and doping values through field-effect measurement, capacitor test structures need to be utilized to measure gate capacitance instead of using theoretical values for the dielectric constant, especially for the thinner gate oxides where quantum capacitance (*C*_q_) of graphene influences the gate capacitance.^35^

In this work, we demonstrate optimization of wafer-scale GFET fabrication and device characterization toward high device yield and uniformity targeted for sensor arrays. The focus is on the GFET processing, including gate, contact, graphene, and encapsulation modules compatible with CMOS BEOL integration. Measurement and analysis of the fabricated GFETs are done on wafer scale with a specific set of test structures with varying device dimensions distributed evenly across the wafer within process control monitor (PCM) areas. The purpose of the PCM characterization is to monitor the quality to ensure stable process conditions so that faulty chips do not proceed further to the characterization of the actual sensor chips. The objective is to extract mobility, contact resistance, transfer-curve hysteresis, and doping of the devices with a minimal number of measurements to develop a protocol for quality control for the GFET fabrication in a pilot-line environment. The work done here further enables the high-yield and uniformity BEOL CMOS integration and quality control of GFETs for applications such as biosensors, gas sensors, and infrared cameras.

## Results and Discussion

### GFET Processing

We present optimization of the GFET fabrication processes including gate, contact, graphene, and encapsulation modules (Figure S1). The cross-section and process parameters of the GFETs are shown in Figures 1a and 1b, respectively. The process is targeted to improve our earlier CMOS BEOL GFET integration process^4^ considering the main BEOL integration limitation with the temperature budget of 400 °C.

**Figure 1:**
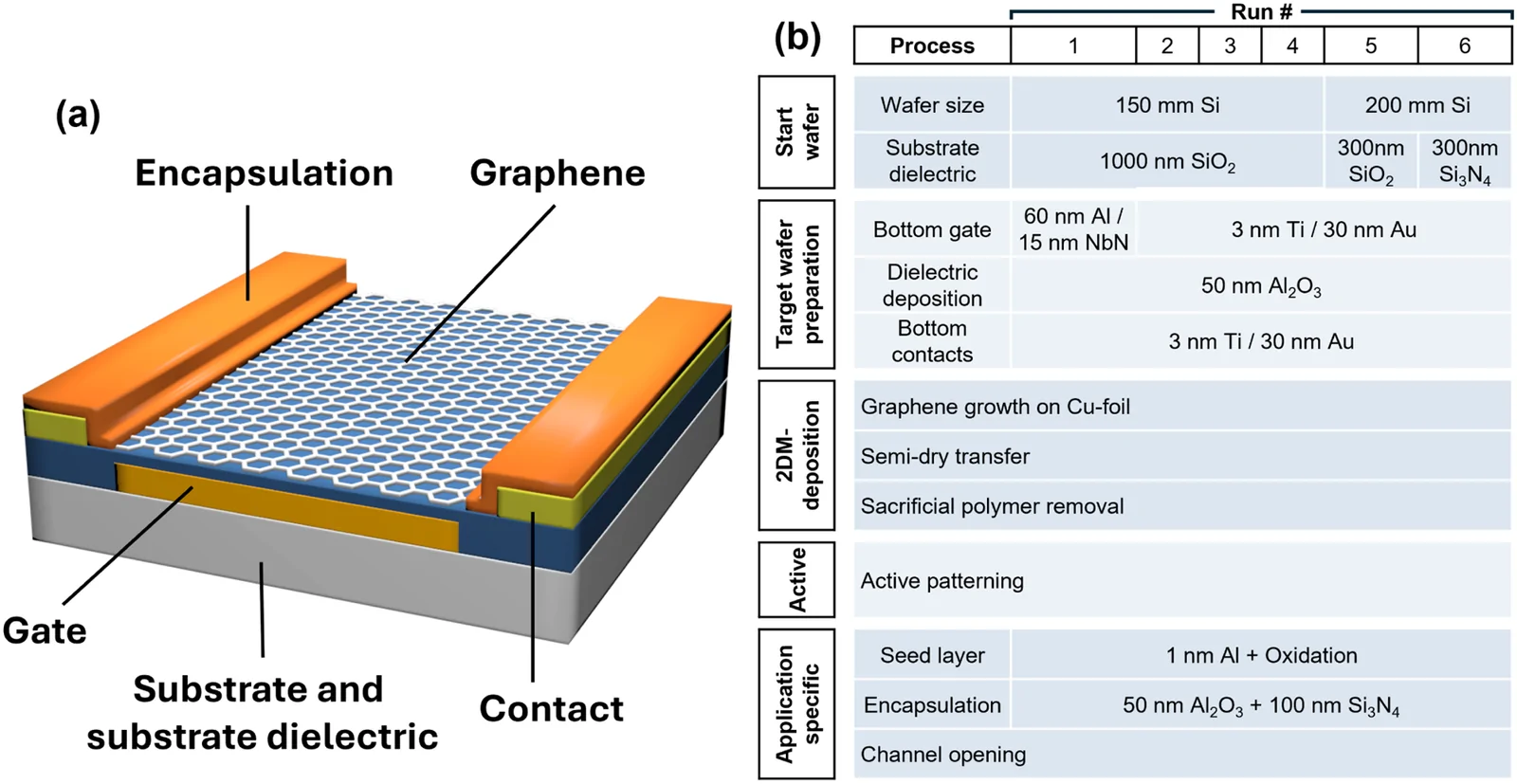
(a) A schematic of the basic modules included in the GFET fabrication process. The modules include back-gate deposition, bottom-contact deposition, graphene transfer, patterning, and encapsulation options. (b) The process flow of the GFET fabrication for Runs 1–6.

The wafer layout consists of different 10 mm × 10 mm sensor chips and identical 10 mm × 2 mm quality-control chips with test structures. A quality-control chip is always located next to a sensor-chip design. A bottom-contact process has been selected to minimize the processing steps after the graphene transfer step. The benefit of this approach is to reduce the residues and simplify the cleaning of the graphene surface. Local back gates are utilized since global back-gating with Si is not possible in the Si CMOS BEOL integration. Overall, 6 different wafer runs have been fabricated to optimize these modules (Runs 1–6). In addition, a final run with 4 wafers has been fabricated to study the difference between one- and two-layer resist lithography for the graphene patterning step (Run 7).

### Test Structures and Analysis Protocols

The performance of the GFETs was characterized by using a semiconductor parameter analyzer, impedance analyzer, and an automated probe station to map out the wafer-scale statistics for each wafer. An example of the set of test structures used is shown in Figure 2. The test structures include GFETs (Figure 2a), gTLMs (Figure 2b), metal-insulator-metal (MIM) and metal-insulator-semiconductor (MIS) capacitors (Figure 2c), and gated CBK (gCBK) resistors (Figure 2d). The resistance of the GFET and gTLM structures is measured for a series of channel lengths (10–50 μm) and widths (5–50 μm). These are analyzed using the gated TLM method, either by Drude formula or by transconductance, to extract both mobility and contact resistance.^26^ We will distinguish between the test structures and analysis method by referring to the prior as gTLMs and the latter as the gated TLM. We also compare the use of DTM, FTM, and Drude methods to the gated TLM (Drude and transconductance).^26^ We chose to use these established extraction approaches that are already widely employed in the GFET literature and to compare their outputs under identical low-field measurement conditions. Detailed validation of mobility-degradation models, such as those shown by Jeppson et al.,^38^ is outside the scope of this comparison. The GFETs and gTLMs include additional leads that enable 4-point Kelvin measurements to remove parasitic resistance. Direct contact resistance measurements are performed by using the gCBK structures.

**Figure 2:**
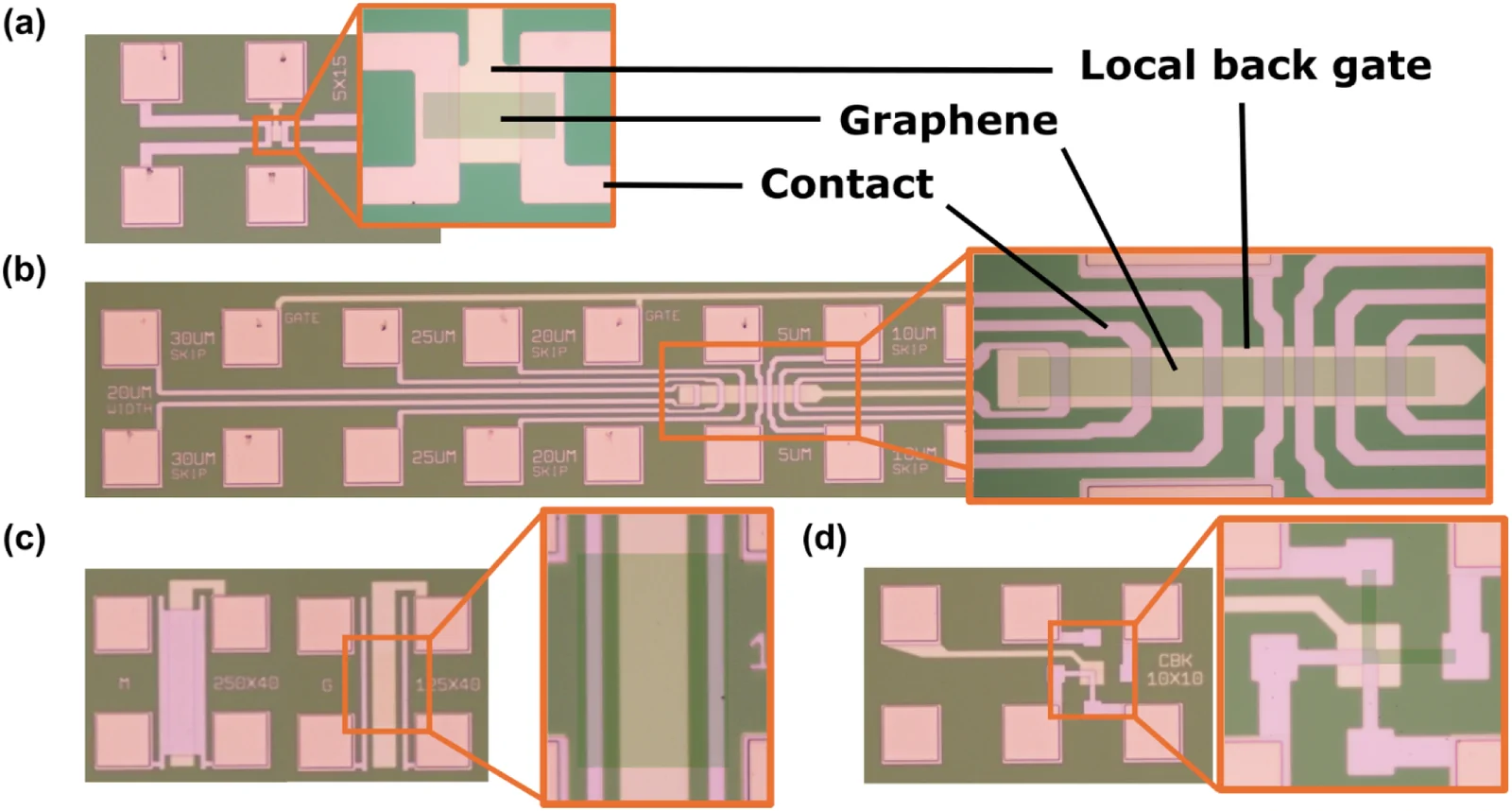
Electrical test structures with (a) a GFET, (b) a gTLM structure, (c) MIM (left) and MIS (right) capacitors, and (d) gCBKs. Inset in (a)–(d) are larger-magnification images with the graphene channel pseudo-colored.

### Back Gate and Bottom Contact Optimization

The contact and back gate roughness, step heights, and discontinuities were characterized by AFM and SEM. In Run 1, the back gate roughness and step height were 1.9 and 77 nm, respectively (Figure 3a). The back gate patterning method was changed from etching to liftoff and material from Al/NbN to Ti/Au after Run 1. This resulted in lower back gate metal RMS roughness of 0.9 nm and step height of 33 nm (Figure 3b). The process change to liftoff resulted in an edge discontinuity presenting as additional edge height of 110 nm in Run 2. In Run 3 (not shown), this was reduced to 60 nm, and to 0 nm in Run 4 (Figure 3c) by optimizing resist undercut in the reverse-bake lithography process. The surface roughness was further optimized in Runs 3–4, finally resulting in a back gate RMS roughness and step height of 0.7 and 33 nm, respectively. Runs 5–7 had similar roughness and step height values as obtained in Run 4 without edge discontinuity issues.

**Figure 3:**
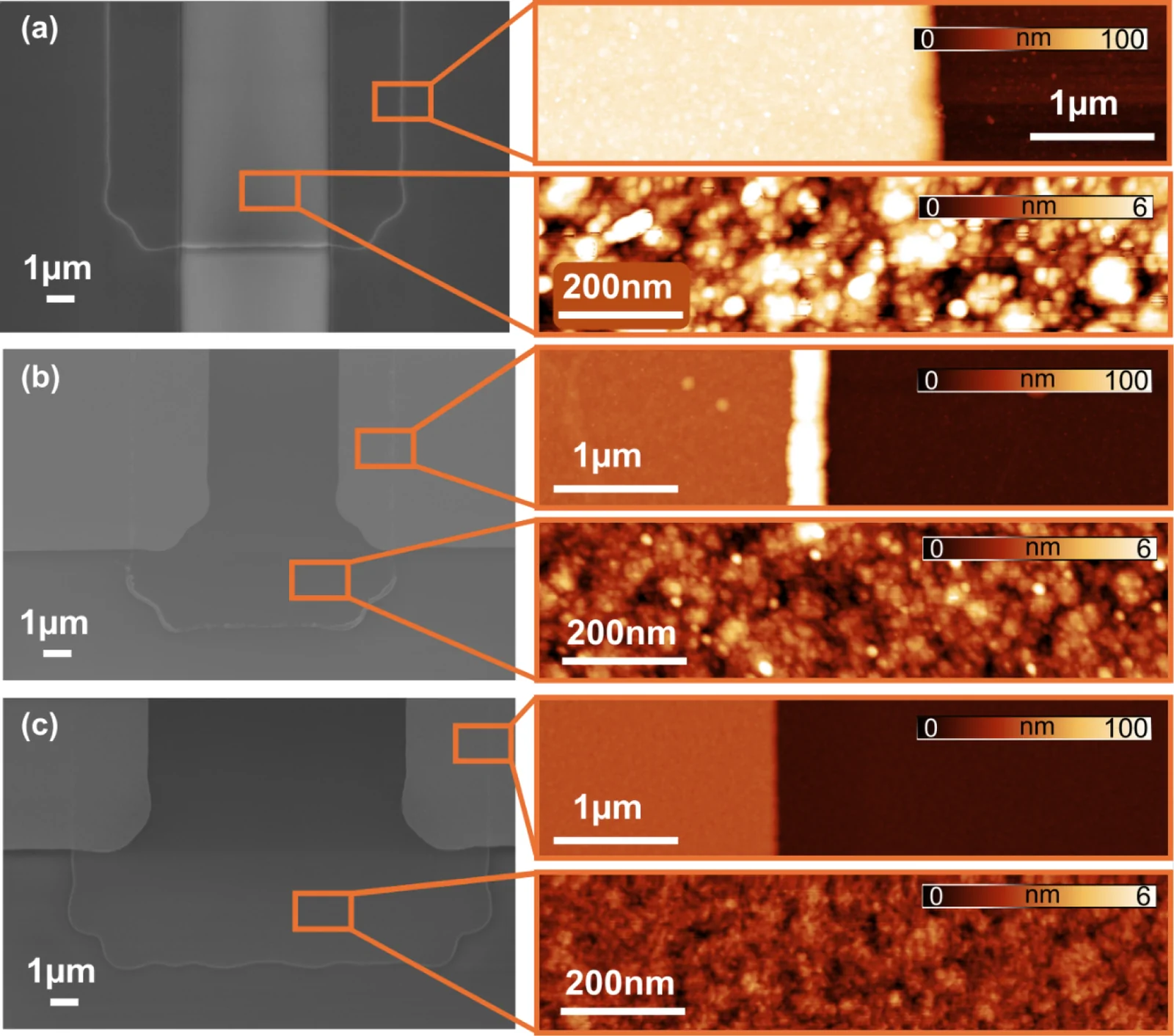
SEM and AFM images of a local back gate and bottom contacts. The inset AFM images depict the gate metal edge (upper) and back-gate metal roughness (lower). (a) Run 1:77 nm back-gate step height and roughness *R*_q_ = 1.9 nm. (b) Run 2:33 nm back-gate step height, roughness *R*_q_ = 0.9 nm, and 110 nm edge discontinuity. (c) Run 4:33 nm back-gate step height, roughness *R*_q_ = 0.7 nm, and no edge discontinuity.

### GFET Test Structure Characterization with Gated TLM

Specific contact resistance, mobility, doping, and yield are extracted for the wafers in Runs 1–6 using eqs 1, 3, and 4, according to the criteria described earlier. For each wafer, a series of GFETs were measured ranging from *L* = 10–50 μm and *W* = 5–50 μm, with a back-gate voltage range of −10–10 V. Individual GFET channels were measured for each width and length, and gated TLM fitting was performed on a set of GFETs with the same width. An example of extracted and normalized transfer curves for a series of GFETs from Run 6 are shown in Figure 4a. The measured transfer curves are symmetrical around *V*_Dirac_, which we typically observe when the devices are passivated and have low doping values. These correspond well to the ones shown earlier with measurements done in vacuum.^25^ Run 6 electrical characterization also included both forward and backward gate-voltage sweeping to study the hysteresis of the devices (Figure 4b).

**Figure 4:**
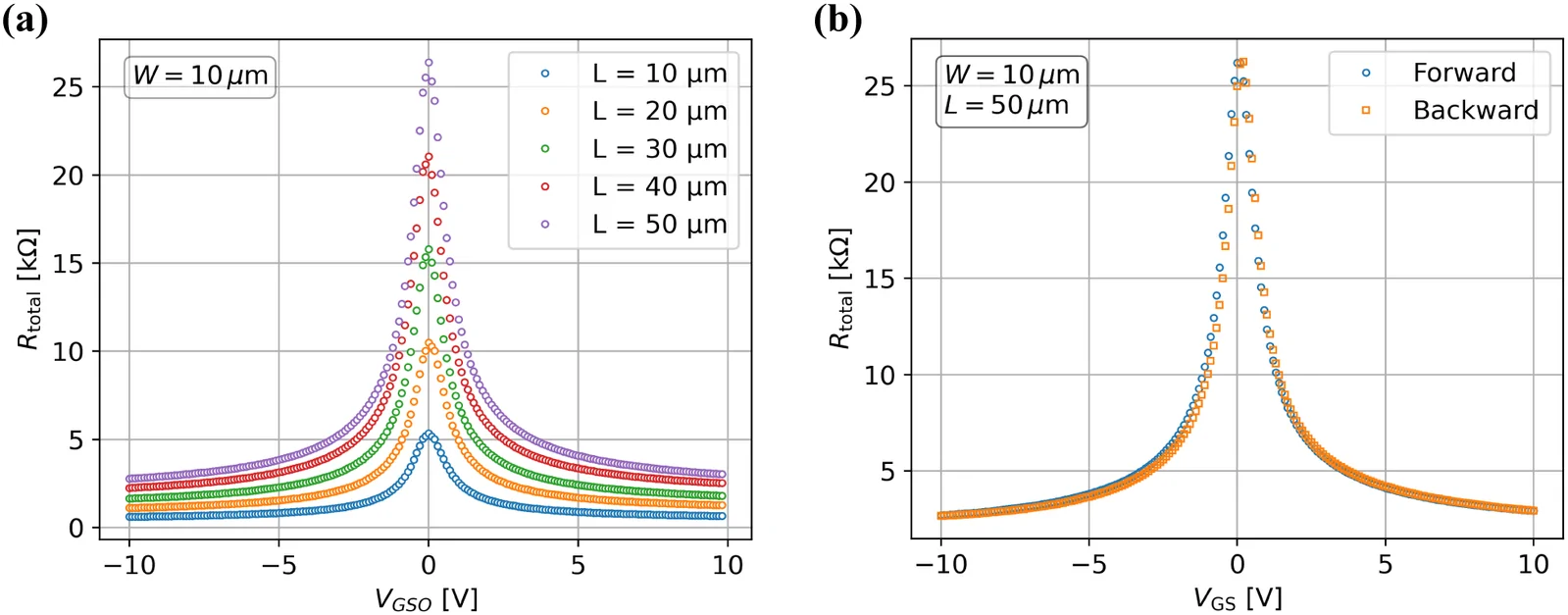
(a) Normalized GFET transfer curves for devices with *W* = 10 μm and *L* = 10–50 μm from Run 6. (b) A GFET transfer curve with both forward and backward transfer curves for a device with *W* = 10 μm and *L* = 50 μm from Run 6.

The wafer-scale statistics for the extracted parameters are shown in Figure 5. For Runs 1–6, the number of GFETs measured for each wafer is 768, 1536, 1536, 1536, 552, and 4320, respectively. The number of devices measured for Run 5 is smaller due to PCM area restrictions on the wafer. The average width-normalized specific contact resistance (*ρ*_c_) values at a carrier concentration of −7.5 × 10^12^ cm^–2^ (Figure 5a) are 1680 (SD = 1050) Ωμm, 760 (SD = 870) Ωμm, 630 (SD = 350) Ωμm, 500 (SD = 320) Ωμm, 400 (SD = 420) Ωμm, and 640 (SD = 310) Ωμm for Runs 1–6, respectively. The data show that Runs 1 and 2 with higher metal step or edge discontinuity heights either on the back gate or on the contacts, had clearly higher specific contact resistivities with higher variations. Runs 3–6 show quite similar specific contact resistance values with similar variations. It seems that graphene can tolerate some edge discontinuity and step heights in the bottom contacts, since the around 60 nm edge discontinuity issues in Run 3 yielded comparable values to Runs 4–6 with around 30 nm back-gate and bottom-contact metal step heights. The data also include nonphysical negative *ρ*_c_ values, which are quite common in large data sets with fitting-based extraction relying on data from several graphene channels due to the variations between the channels, such as fabrication defects, including, for example, bilayer regions and holes, as well as different graphene sheet resistance values under the metal and channel areas^25,32–34,39,40^ Another possible reason for the negative contact resistances can be due to contact-induced channel doping.^41^ Correcting the channel dimensions to the actual values measured by SEM reduced the amount of negative values and overall variation of the values. Yield criteria could also be further evaluated to allow narrower device variation, which could eliminate the extraction of negative values.

**Figure 5:**
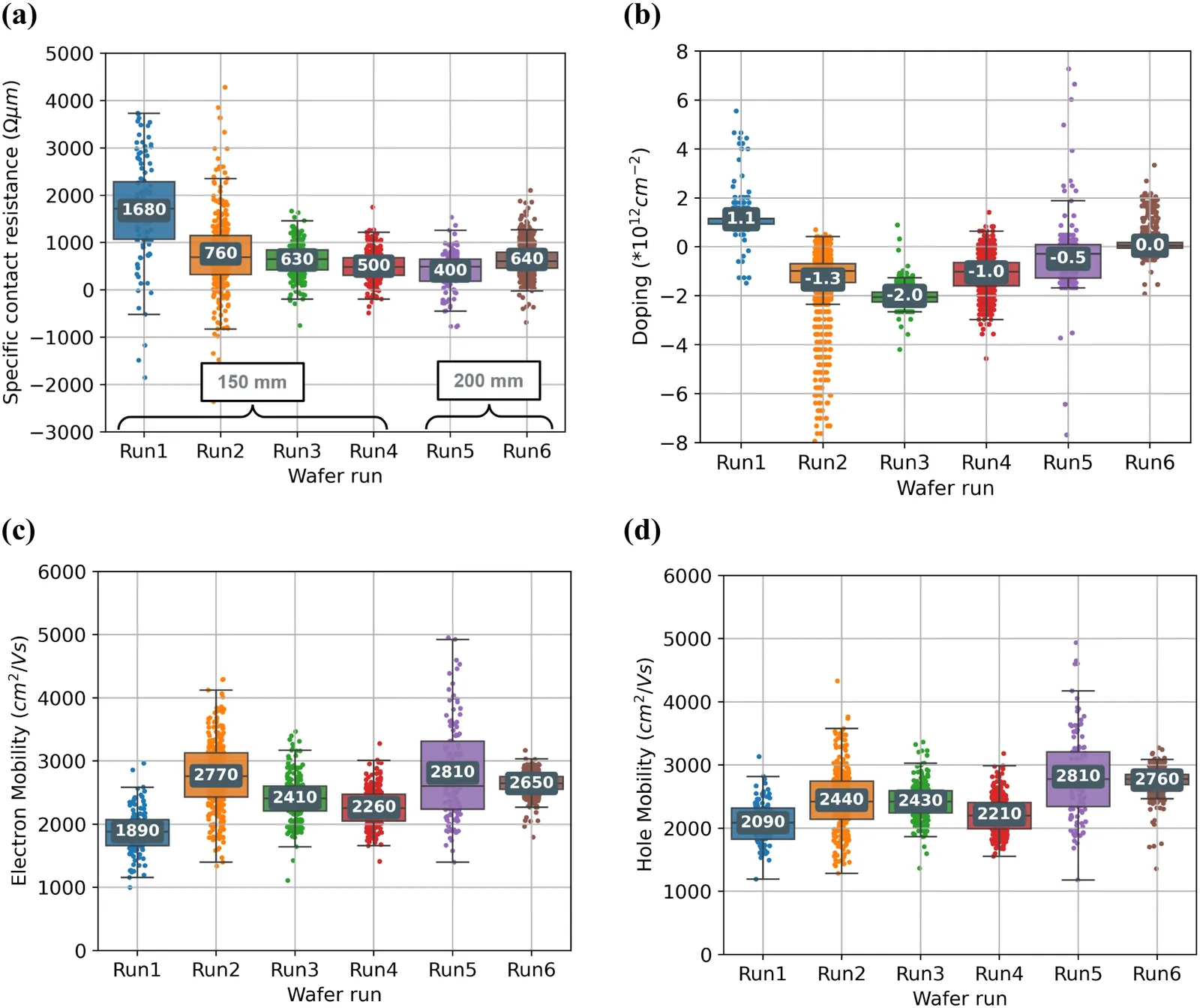
Statistical analysis of process Runs 1–6 with electrical measurements for (a) specific contact resistance at a carrier concentration of −7.5 × 10^12^ cm^–2^, (b) doping, (c) electron mobility at a carrier concentration of 1 × 10^12^ cm^–2^, and (d) hole mobility at a carrier concentration of −1 × 10^12^ cm^–2^. Inset values are average values of each wafer.

The average doping values (Figure 5b) range from −2.0 to 1.1 (SD ≈ 1) × 10^12^ cm^–2^ for Runs 1–6. There is no clear trend visible in the doping values, and the values are varying slightly in both negative and positive carrier concentrations. For average electron mobility $\left(\mu_{e}^{\mathrm{TLM}}\right)$ (Figure 5c) and hole mobility $\left(\mu_{h}^{\mathrm{TLM}}\right)$ (Figure 5d), we observe an upward trend starting from the average value of 1890 (SD =350) cm^2^/(V s) for electrons and 2090 (SD = 340) cm^2^/(V s) for holes in Run 1 and ending in an average value of 2650 (SD = 150) cm^2^/(V s) for electrons and 2760 (SD = 160) cm^2^/(V s) for holes in Run 6, respectively. We believe this increase is likely due to the decrease in the roughness on the gate stack area, as also observed recently in comparisons between recessed and raised gate structures.^42^ The GFET device yields of Runs 1–6 are 88%, 79%, 99%, 99%, 93%, and 97%, respectively. Despite having a large back gate step height and *R*_q_, Run 1 shows a good device yield but clearly reduced electrical performance. The significant edge discontinuity in Run 2 (Figure 4b) caused a significant drop in yield (predominantly due to open circuits), which, when partially resolved in Run 3, led to a significant improvement in yield.

The values obtained in Run 6 are also shown as wafer maps in Figure 6. Average ρ_c_ (Figure 6a) in the wafer is 640 (SD = 310) Ωμm. The average $\mu_{h}^{\mathrm{TLM}}$ (Figure 6c) is 2760 (SD = 160) cm^2^/(V s). The average intrinsic doping (Figure 6b) value for all measured GFETs in the wafer is 0.0 × 10^12^ (SD = 0.3 × 10^12^) cm^–2^. The average hysteresis (Figure 6d) value with 20 V measurement range is −0.4 (SD = 0.9%).

**Figure 6:**
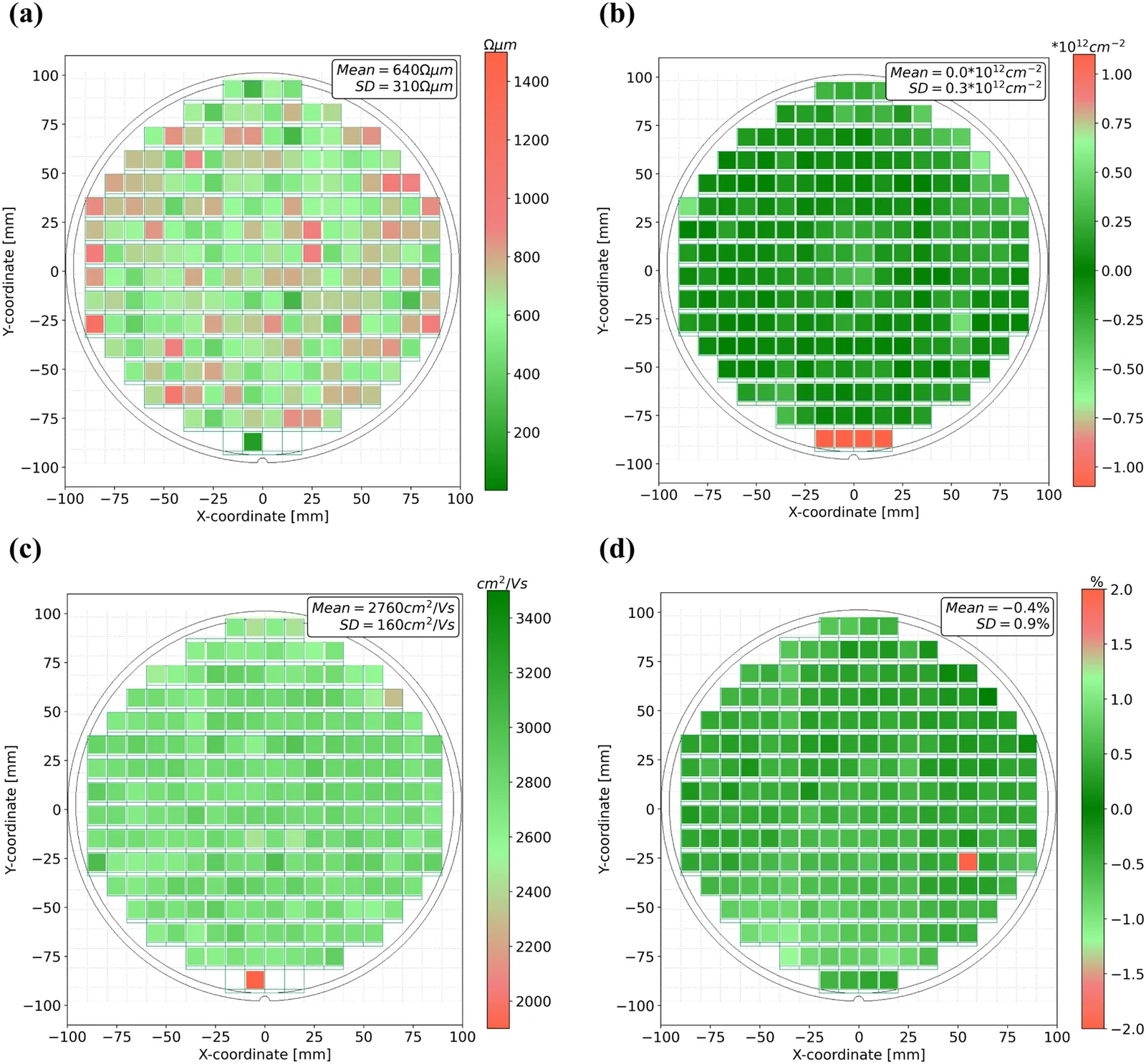
Wafer maps of process Run 6 with electrical measurements for (a) specific contact resistance at a carrier concentration of −7.5 × 10^12^ cm^–2^, (b) doping, (c) hole mobility at a carrier concentration of −1 × 10^12^ cm^–2^, and (d) hysteresis. Inset values are for the mean and standard deviation of each parameter.

### Comparison between GFETs and gTLM Structures with the Gated TLM

A comparison between GFETs and gTLM test structures was conducted in Run 6 by analyzing both with the gated TLM using eqs 1, 3, and 4 to extract doping, specific contact resistance, and hole and electron mobility values. This is shown in Figure 7. The measurements with GFETs and gTLMs were repeated twice, with a 6-month delay after the first measurement session. total of 1298 gTLM channels (6 lengths per gTLM, 216 individual gTLMs total) with *L* = 5–30 μm and *W* = 10 and 20 μm were measured in addition to the 4320 GFETs each time.

**Figure 7:**
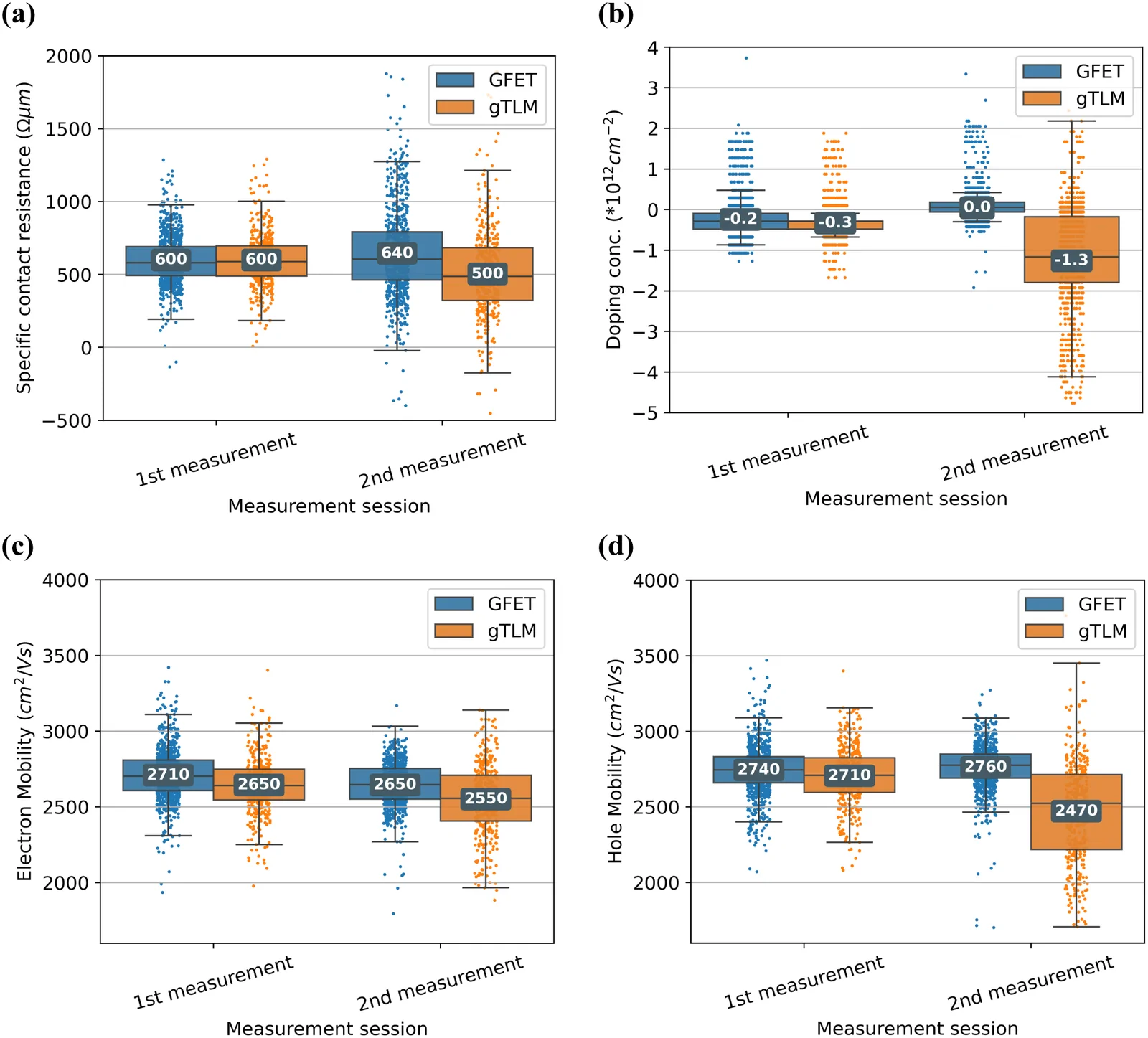
Statistical analysis of Run 6 comparing GFET and gTLM test structures with (a) specific contact resistance at a carrier concentration of −7.5 × 10^12^ cm^–2^, (b) doping, (c) electron mobility at a carrier concentration of 1 × 10^12^ cm^–2^, and (d) hole mobility at a carrier concentration of −1 × 10^12^ cm^–2^. GFETs are indicated in blue and gTLMs in orange. Inset values are average values of each dataset.

The average values and standard deviations for doping, specific contact resistance, and hole and electron mobility are shown for the GFETs and gTLMs in Figure 7. The average contact resistance values (Figure 7a) for the first round of measurements yielded similar values of 600 (SD = 170) Ωμm and 600 (SD = 190) Ωμm for GFETs and gTLMs, respectively. In the second round, the values were 640 (SD = 310) Ωμm and 500 (SD = 340) Ωμm for GFETs and gTLMs, respectively. The results were still quite similar, with standard deviation increasing for the second round of measurements, with gTLMs having a slightly lower average. The average doping values (Figure 7b) are −0.2 × 10^12^ (SD = 0.3 × 10^12^) cm^–2^ and 0.0 × 10^12^ (SD = 0.3 × 10^12^) cm^–2^ for GFETs and −0.3 × 10^12^ (SD = 0.3 × 10^12^) cm^–2^ and −1.3× 10^12^ (SD = 1.4 × 10^12^) cm^–2^ for gTLMs across the two measurement sessions, respectively. The average electron mobility values were also quite constant for the GFETs, showing values of 2710 (SD = 170) cm^2^/(V s) for the first round and 2650 (SD = 150) cm^2^/(V s) for the second round. The electron mobility values for the gTLMs showed a larger difference between the measurement rounds, with average values of 2650 (SD = 190) cm^2^/(V s) for the first round and 2550 (SD = 240) cm^2^/(V s) for the second round. The average hole mobility values also continued the same trend, with GFETs having more stable behavior, with the average values of 2740 (SD = 170) cm^2^/(V s)) for the first round and 2760 (160) cm^2^/(V s) for the second round, while the gTLM structures yielded 2710 (SD = 200) cm^2^/(V s) for the first round and 2470 (SD = 340) cm^2^/(V s) for the second round. These measurements show clearly that the GFETs give much more repeatable results with smaller variation when compared to the gTLM structures. This effect can be strongly observed from doping to a lesser extent from mobility. The small changes in the GFET results are likely due to time and the samples being stored in ambient conditions despite being protected by passivation. The original condition of the samples can likely be restored by a vacuum annealing step. The larger changes and overall worse results with gTLM structures cannot be only due to the time between the measurement sessions. Another factor that can contribute to the worse gTLM results is the combined gate electrode for a set of channel lengths, as well as reused source and drain electrodes for certain channel lengths (Figure 1b). Repeated biasing on the same piece of graphene can lead to interference between the different channel lengths being measured, resulting in larger variation in device parameters. This is especially visible from the large change in doping variation in the gTLM structures observed in the second measurement session (Figure 7b).

### Comparison between Mobility and Contact Resistance Extraction Methods

A comparison of mobility extraction methods is shown in Figure 8a for hole mobility extracted from a series of GFETs across the wafer from Run 6. The methods shown are the gated TLM, FTM, Drude and DTM methods, and Hall measurements. In addition, we show the gated TLM utilizing transconductance defined in eq 6. For DTM, the hole mobility is taken from a carrier concentration of −1 × 10^12^ cm^–2^ to be comparable with the other methods. Extracting the hole mobility from this carrier concentration with the DTM does not result in a significant difference in values when compared to the ones extracted at transconductance maximum with our devices (Figure S4). The Hall measurements were performed on individual chips from the same wafer. The total number of GFET channels measured is 4320, and for Hall measurements, 7 chips were randomly selected with 5 repeat measurements on each chip.

**Figure 8:**
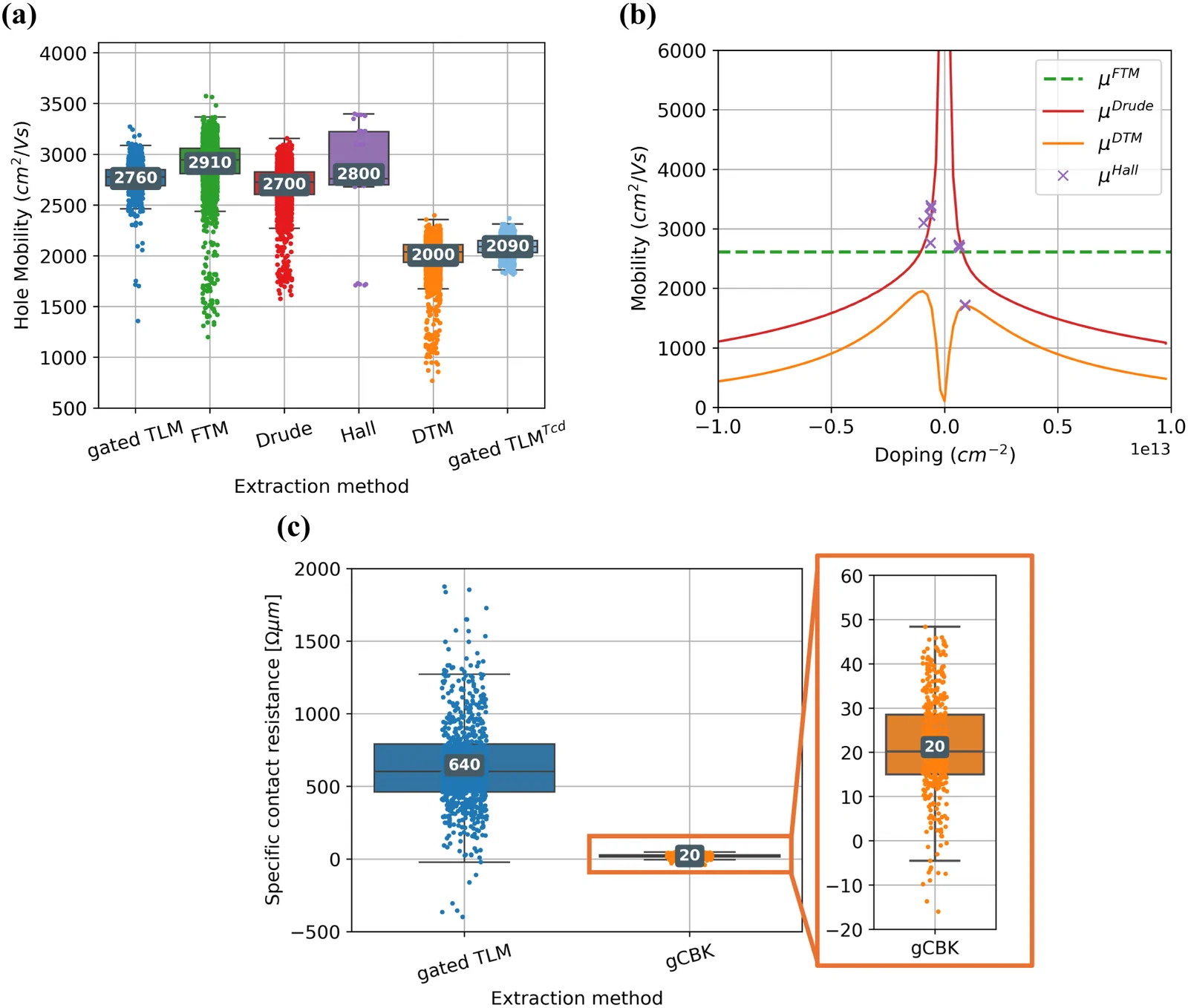
Comparison of the different methods for graphene parameter extraction. (a) Hole mobility from the different extraction methods. For gated TLM, Drude, and DTM methods, the mobility is extracted at a carrier concentration of −1 × 10^12^ cm^–2^. gated TLM^Tcd^ is the gated TLM using the transconductance equation. There are 35 data points for Hall measurement (7 chips). (b) Mobility of selected methods against doping concentration. (c) Specific contact resistance from the gated TLM and gCBK test structures. Inset is a magnified view of the gCBK specific contact resistance. Inset values are average values of each method.

The gated TLM, FTM, and Drude methods result in very similar average hole mobility values of 2760 (SD = 160) cm^2^/(V s), 2940 (SD = 240) cm^2^/(V s), and 2700 (SD = 190) cm^2^/(V s), respectively. These methods are different implementations of the Drude model. The gated TLM and FTM aim to separate the contribution of *R*_c_, which results in a higher *R*_sh_, and thus higher μ_h_. The FTM additionally considers the real, nonideal characteristics of GFETs around the Dirac peak by including the *n*_0_ term, which is needed to stabilize the mobility plot near *V*_dirac_. FTM also produces only one mobility value per device and not carrier concentration-dependent mobility values as the other two methods. Hall measurements show an average mobility of 2800 (SD = 510) cm^2^/(V s), which is close to the values obtained by the previous three methods. The Hall measurements were done without gating, and so the doping concentration is only measured and cannot be controlled. The average hole mobility values for the DTM and transconductance-based gated TLM (gated TLM^Tcd^) are close to each other, ranging from 2000 (SD = 170) cm^2^/(V s) to 2090 (SD = 90) cm^2^/(V s). The primary difference is that the DTM does not consider *R*_c_. Drude, DTM, and FTM methods, which extract mobility from single devices, show dependence on device lengths, with larger channels producing higher mobility values, most likely due to the contact resistance either not being extracted at all or not being extracted correctly. The methods show either no or weak dependence on the width when compared to the length. See Supplementary Information Section 3 for more details.

The Drude model-based methods produce significantly higher mobility values than the transconductance-based methods (DTM and gated TLM^Tcd^). The most significant difference between these two groups of methods is that transconductance (eq 6) defines mobility using the derivative $\left(\partial \sigma_{s}h\right)/\left(\partial V_{G}SO\right)$ ­(∂σ*_s_h*)/(∂*V*_G_ *SO*). This form of the equation neglects the contribution of gate induced mobility modulation in the sheet conductance derivative, and other derivatives (see Supplementary Information Section 3 for more details). Plotting the mobility extracted from these methods against doping concentration (Figure 8b), we find that the Hall measurement results support the use of the Drude model to extract mobility.

The specific contact resistance values obtained from the 5 μm × 5 μm and 10 μm × 10 μm gCBK structures in Run 6 are shown in Figure 8c and compared to *ρ*_c_ from the gated TLM. *R*_c_ values calculated according to eq 9 were normalized with the contact width using eq 10. 432 gCBKs were measured in total. The *R*_c_ values for the 5 μm × 5 μm and 10 μm × 10 μm CBK structures are 4 (SD = 3) Ω and 2 (SD = 1) Ω, respectively. The *ρ*_c_ values obtained by normalizing with the width of the devices for the 5 μm × 5 μm and 10 μm × 10 μm CBK structures are 20 (SD = 14) Ωμm and 22 (SD = 10) Ωμm, respectively (Figure S6a). The average and standard deviation values are clearly smaller when compared to the values extracted from the GFETs or gTLMs with the gated TLM. Similar values are obtained with gCBKs when the data are normalized by the device width. However, further analysis of the specific contact resistance values extracted from the GFETs using the gated TLM reveals a clear width dependency. Specifically, devices with 5 μm wide channels exhibit a higher average specific contact resistance of 900 (SD = 320) Ωμm, which decreases with increasing channel width, reaching 460 (SD = 250) Ωμm for 50 μm wide channels (Figure S6b), at a carrier concentration of −7.5 × 10^12^ cm^–2^. In addition to this, the carrier concentration dependency was studied for both test structure types. GFETs (Figure S7a) with gated TLM yield *ρ*_*c*_ values of 640 (SD = 310) Ωμm at a carrier concentration of −7.5 × 10^12^ cm^–2^ and 2470 (SD = 1810) Ωμm at the Dirac point. The values extracted with gCBK structures (Figure S7b) show *ρ*_*c*_ values of 21 (SD = 14) Ωμm at a carrier concentration of −7.5 × 10^12^ cm^–2^ and 42 (SD = 13) Ωμm at the Dirac point. Values extracted from GFETs exhibit clearly high dependency on the carrier concentration and higher variation at the Dirac point when compared to the values from gCBKs. Due to this high variation around the Dirac point with the gated TLM, we consider that the higher carrier concentration values are more reliable for the extraction of the specific contact resistance values with the method. For both structures, one needs to consider that the gating with the bottom gate and bottom-contact device configuration does not actually gate the contact in the area where metal and graphene overlap, and the impact is coming from the gating of the graphene channel area.

The large differences between the GFETs (gated TLM) and gCBKs regarding the specific contact resistance values and different dependencies on channel width and carrier concentration indicate that these test structures are measuring different factors contributing to the total specific contact resistance of the device. These methods should be seen as complementary to each other, as shown by König et al.^33^ when using a modified CBK structure to measure contact interface resistance. Together with this and contact resistance extracted from TLM, the specific area contact resistivity *ρ*_c,A_, transfer length *L*_T_, and graphene sheet resistance *R*_sk_ can be extracted. Average contact resistance and interface resistance values extracted from the 10 μm-wide gated TLM and gCBK devices at a carrier concentration of −7.5 × 10^12^ cm^–2^ have also been utilized to extract specific area contact resistivity *ρ*_c,A_, transfer length *L*_T_, and graphene sheet resistance *R*_sk_ at the contact. The values for *ρ*_c,A_, *L*_T_, and *R*_sk_ are 1.5 × 10^–5^ Ωcm^2^, 2.4 μm, and 250 Ω / □, respectively. *R*_sk_ value indicates that there is different doping on the graphene on top of the contact than on the channel area, where the comparable sheet resistance value *R*_sh_ is 580 Ω / □. The extracted transfer length of 2.4 μm indicates efficient current injection from graphene into the metal, with most of the current transferring within a few micrometers from the contact edge. Since the contact length (10 μm) is significantly larger than *L*_T_, the contacts are effectively utilized and are not strongly limited by current crowding. Furthermore, the specific area contact resistivity of 1.5 × 10^–5^ Ωcm^2^ confirms a reasonably low-resistance metal–graphene interface and indicates good contact quality, although additional reductions in *ρ*_c,A_ would further improve current injection and reduce the overall contact resistance.

Gated TLM measurements are also impacted by the sheet resistance variations between the different channels and not considering the difference in the sheet resistance in the channel and at the metal contact area.^25,32–34,39,40^ In addition to the modified CBK structures, there are 3-point CER structures which have been presented to provide complementary information to the traditional TLM structures.^25,32–34^ The impact of metal lead resistances^27,43^ was negated in the TLM results by utilizing 4-point measurements. FTM was also used to estimate *ρ*_*c*_, resulting in an average value of 5740 (SD = 2540) Ωμm (not shown in Figure 8c) which is much larger compared to the other methods. This is likely due to FTM failing to properly separate mobility and contact resistance parameters by trying to simultaneously extract multiple parameters by fitting.^44^ Due to this, FTM should be used with caution.

### One-Layer and Two-Layer Resist Approach for Graphene Patterning

The combined layer thicknesses of graphene and polymer residues for the Run 7 wafers were measured by AFM after the graphene patterning step for the one- (Figure 9a) and two-layer (Figure 9b) patterning approaches. For each wafer, 5 locations across the wafer have been measured (Figure 9c), giving us average thicknesses of 2.5 nm (SD = 0.2 nm), 2.3 nm (SD = 0.4 nm), 1.0 nm (SD = 0.1 nm), and 1.3 nm (SD = 0.2 nm) for wafers W1, W2, W3, and W4, respectively. This indicates a clear reduction in resist residues with the utilized two-layer approach, enabling cleaner graphene surfaces required, for example, for functionalization.

**Figure 9:**
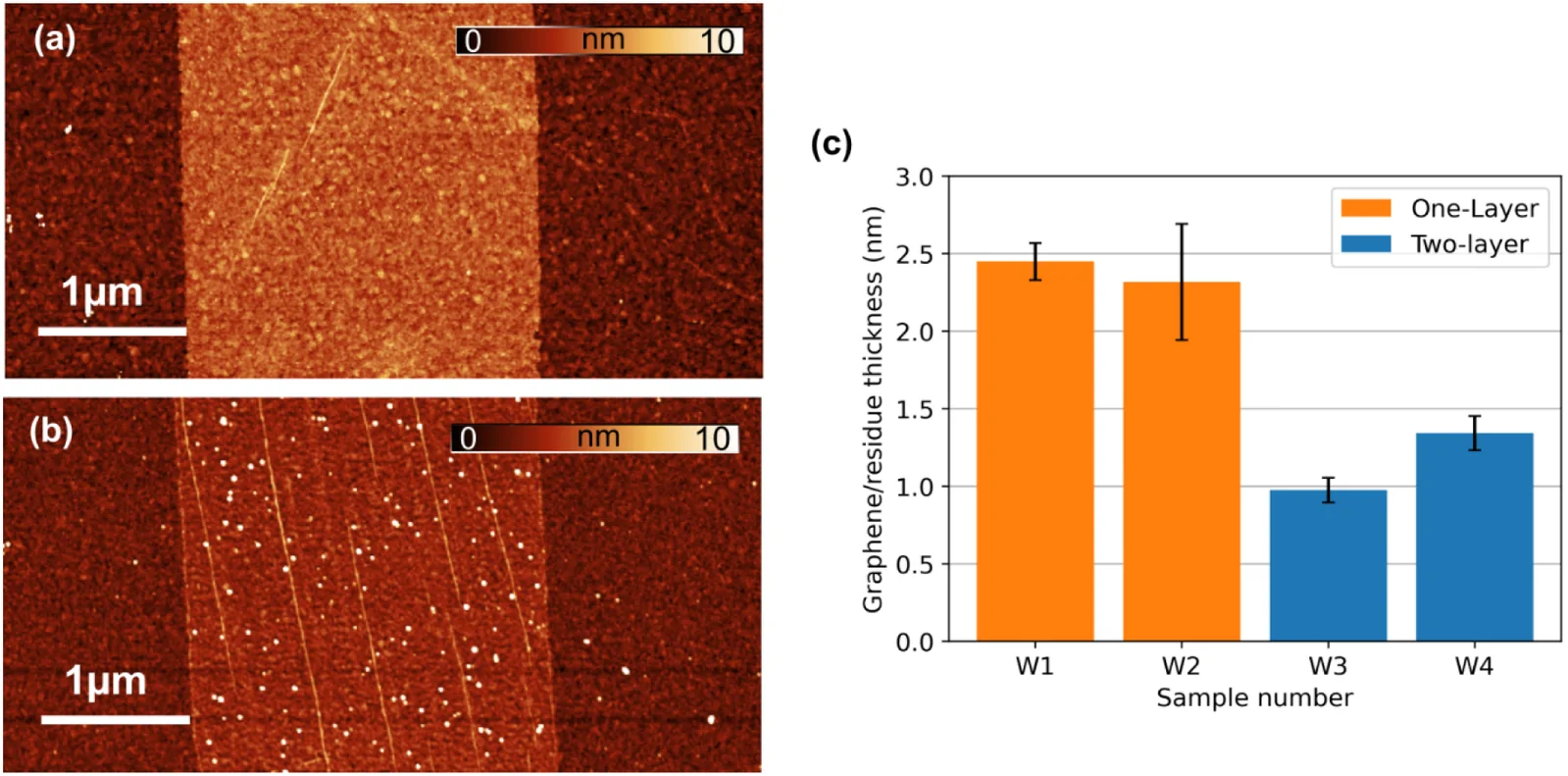
(a, b) Example AFM scans of the GFET channel area used to measure the combined thickness of graphene and resist residues. Graphene patterning was done with (a) one-layer and (b) two-layer resist patterning. (c) The average graphene and resist thickness and standard deviation values for 4 different wafers with one- and two-layer resist patterning. One- and two-layer patterning is shown in orange and blue, respectively.

The wafers of Run 7 were electrically characterized to determine the effect of residues on the doping and uniformity of GFETs. The doping values calculated by using eq 3 for W1 (Figure 10a) and W2 (Figure 10b) with one-layer resist patterning, and for the W3 (Figure 10c) and W4 (Figure 10c) with two-layer resist patterning, showed average values of 18.4 (SD = 3.8) × 10^12^ cm^–2^, 12.9 (SD = 2.4) × 10^12^ cm^–2^, 11.6 (SD = 1.4) × 10^12^ cm^–2^, and 9.3 (SD = 1.2) × 10^12^ cm^–2^, respectively. These show a clear reduction with the two-layer patterning approach in overall doping, as well as an improvement in doping uniformity across the wafer, which are very beneficial for the wafer-scale processing of GFET sensor arrays. These values have been obtained with non-passivated devices in ambient conditions, which has an impact on the overall doping as well as the standard deviation values. This is not optimal for quality control devices but has been used to test the expected doping values for actual sensor arrays operated in ambient conditions. These measurements reveal that sensor characterization also in ambient conditions can provide additional insights into the process uniformity and cleanliness of the sensor surface. Comparing these results to those seen in Figure 6b, device passivation improves doping uniformity even with the one-layer patterning approach, but also hides the non-uniformity caused by the resist residues. Due to the wider doping spread of the devices caused by ambient conditions, lack of passivation layers, and different dielectric stack, Run 7 is not compared to the other runs with parameters such as mobility and contact resistance.

**Figure 10:**
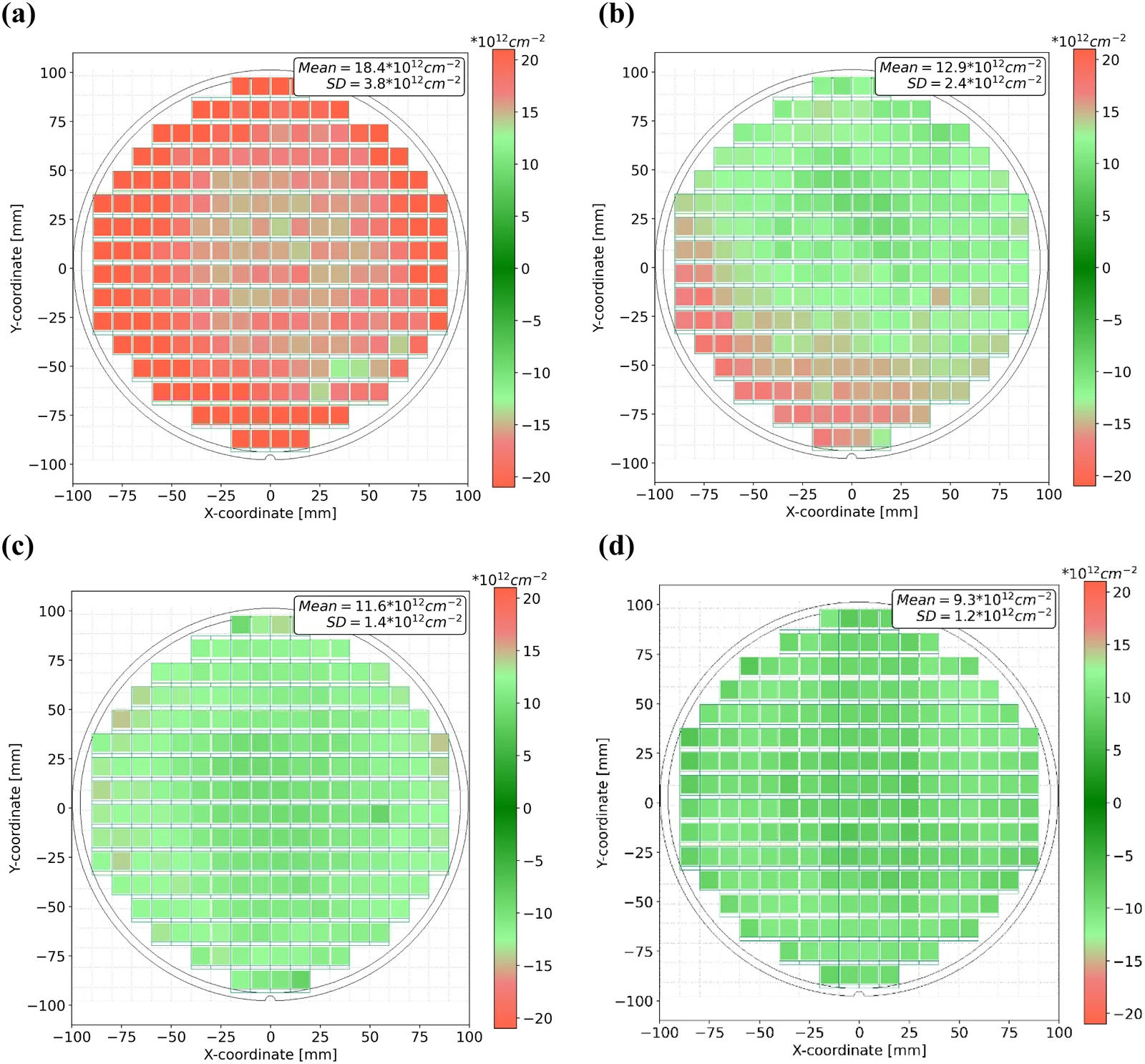
Wafer maps of doping concentration measured electrically for Run 7, utilizing one-layer resist for wafers (a) W1 and (b) W2, and two-layer resist for wafers (c) W3 and (d) W4 in graphene patterning.

## Conclusions

We demonstrate a high-yield and uniform CMOS BEOL-compatible GFET fabrication process, including the gate, contact, graphene, and encapsulation modules, as well as statistical analysis comparing different encapsulated test structures and analysis methods for the extraction of mobility, contact resistance, doping, and transfer-curve hysteresis values. The fabrication process and quality control protocols can be utilized, for example, in the wafer-scale fabrication of CMOS-integrated GFET-based biosensors,^4^ gas sensor arrays,^12^ and infrared cameras^11^ that have been reported in the past. When compared to the previously published devices with local back gates targeted for sensor devices with the same graphene source, we can see an improvement in mobility, contact resistance, and transfer-curve hysteresis values, which is likely due to the more optimized bottom-gate stack and device passivation.^2,9^

The process optimization done in the process runs shows the impact of back-gate roughness and back-gate and contact metal step heights on the electrical performance of the devices. Overall, the roughness and metal step heights should be minimized to optimize device performance. The comparison done between one- and two-layer resist-patterned graphene channels shows a clear difference in the thickness of the polymer residue layer as well as in the overall doping and wafer-scale uniformity values. The reduction of the polymer residue layer remaining on top of the graphene after device fabrication can be critical for the subsequent graphene-specific functionalization steps, for example, in biosensing applications.^45^

Comparison between different test structures and analysis methods also clearly shows that the selected approach can have a large impact on the values and repeatability of the measurements. GFETs analyzed with the gated TLM show better wafer-scale uniformity for mobility, specific contact resistance, and doping values when compared to gTLM structures. This is especially clear when conducting repeat measurements, with the gTLM structures showing larger variation and degradation over two measurement sessions, whereas the GFET values were much more reproducible and stable. The observed differences between the values obtained from different test structures and analysis methods must be taken into account when comparing the values between different sources. The extracted mobility values from GFETs also depend on the selected analysis protocols as shown with the comparison between gated TLM (Drude and transconductance), DTM, FTM, and Drude methods. Methods that do not consider contact resistance are also more sensitive to the channel dimensions, and this is especially critical when measuring smaller devices. Contact resistance values show even greater differences between the gated TLM, gCBK, and FTM approaches. The lowest values are obtained with direct measurement using the gCBK structures. The gated TLM approach also yields relatively low contact resistance values, while the FTM approach shows much higher values, which is likely an artifact caused by the method’s failure to extract several parameters at the same time by fitting.^44^ It needs to be considered that the gCBK and gated TLM approaches extract different contributions to contact resistance and should not be directly compared. The interfacial contact resistance obtained from the gCBK structures, when used together with the gated TLM approach, can be used to obtain additional information about the quality of the contacts. These aspects seen with different test structures and methods need to be considered when comparing the values of the electrical parameters of GFETs from different sources.

Further improvement of the measurement routines could benefit from the reduction of measurement and analysis complexity by using 4-point test structures, such as Hall bar or Van der Pauw structures, to directly measure sheet resistance for mobility extraction in cases where contact resistance is not a critical parameter. With larger device sizes, it is also possible to utilize Drude, FTM and DTM methods directly with GFETs. Using the gated TLM approach (GFETs) together with either gCBK or CER structures is the best option for detailed characterization of the devices when contacts are critical, but this requires the measurement of at least 3–5 graphene channels and one CBK or CER device for the analysis at each location. The gated TLM approach still suffers from some nonphysical negative *ρ*_c_ values obtained in the analysis. These are likely related to channel-to-channel variations and extraction uncertainties.^25,32–34,39,40^ Another possible reason for the negative contact resistances can be due to contact-induced channel doping.^41^ SEM-based dimension correction reduced their occurrence and overall variability, while tighter yield criteria could further improve extraction accuracy and consistency. For industrially relevant usage, the number of measured test structures per wafer also needs to be further limited relative to the quite extensive measurements shown here.

## Methods

### GFET Processing

150 mm Si wafers with 1000 nm thermal SiO_2_ were used as the starting substrates for Runs 1–4. Runs 5–6 were done on 200 mm Si wafers, with 300 nm thermal SiO_2_ for Run 5 and 300 nm LPCVD Si_3_N_4_ for Run 6. The first process step includes the local back gate 60 nm Al/15 nm NbN deposition and dry etching for Run 1, and 3 nm Ti/30 nm Au metal deposition by evaporation and liftoff for the following 5 runs (Runs 2–6). The reverse bake lithography process with AZ5214 was optimized between Runs 2 and 4 to ensure good liftoff profiles with suitable resist undercuts. The gate dielectric of 50 nm Al_2_O_3_ for Runs 1–6 was deposited by atomic layer deposition (ALD). The bottom contact 3 nm Ti/30 nm Au deposition was done by a liftoff process, and graphene transfer was done with a semi-dry transfer process by Graphenea. The graphene was patterned with photolithography and O_2_ plasma dry etching. The devices were encapsulated by first evaporating a 1 nm Al seed layer that was oxidized, followed by 50 nm ALD Al_2_O_3_ and an additional 100 nm PECVD Si_3_N_4_ layer. Finally, the encapsulation was opened by dry etching the Si_3_N_4_ and wet etching the Al_2_O_3_ from the probe pads and channels of the sensor devices, but not from the channels of the quality-control devices used to extract the electrical parameters.

The process flow for run 7 for the final 4 wafers was slightly modified from the run 6 process to study the impact of one- and two-layer resist lithography on the amount of resist residues on the patterned graphene channel and on doping uniformity. In addition, the gate dielectric was changed to 17 nm Al_2_O_3_ and 3 nm HfO_2_. The target of these wafers is to develop a biosensing-compatible process flow where a highly selective channel-opening etch is possible and the surface cleanliness of graphene is improved. Graphene patterning was done for 2 wafers with a one-layer approach with AZ5214 and for 2 wafers with a two-layer approach similar to the solution by Gao et al.^46^ with polydimethylglutarimide (PMGI) and AZ5214. These devices were not encapsulated in this study.

### Wafer-Scale Electrical Characterization

The GFETs and gTLMs were analyzed in a similar manner. A constant drain-source bias of 100 mV is applied, and the total resistance *R*_total_ is measured as a function of gate voltage *V*_GS_. The transfer curves are normalized to the Dirac peak voltage (*V*_Dirac_). Linear fitting is then used to extract sheet and contact resistance as a function of gate-voltage overdrive (*V*_GSO_ = *V*_GS_ – *V*_Dirac_). *R*_total_ consists of channel resistance (*R*_channel_) from graphene and contact resistance (*R*_c_) from the graphene/metal junction. It can be expressed as

1 $$R_{\mathrm{total}}=2R_{c}+R_{\mathrm{channel}}=2R_{c}+\frac{R_{sh}}{W}L$$

where *R*_sh_, L, and W are the sheet resistance, length, and width of graphene. By linear fitting *R*_total_ of graphene channels with width *W* and different *L* at each *V*_GSO_ point, *R*_sh_ and *R*_c_ can be extracted from the slope and intercept, respectively, and the *V*_GSO_-dependent *R*_sh_ and *R*_c_ curves can be obtained.

Mobility as a function of gate voltage is calculated by using the extracted sheet resistance according to the Drude model:^47^

2 $$\sigma_{sh}=\frac{1}{R_{sh}}=\mu en$$

where *e* is the elementary charge, 1.6 × 10^–19^ C, and *n* is the gate-induced charge carrier or doping concentration, which can be formulated with the measured gate capacitance per unit area, *C*_g_:

3 $$n\left(V_{\mathrm{GSO}}\right)=\frac{1}{e}\int_{0}^{V_{\mathrm{GSO}}}C_{g}\left(V\right)dV$$

The gate capacitance value is measured using an impedance analyzer from the capacitor test structures for each wafer run to obtain more accurate mobility and doping values (see Supporting Information Section 2).

The gated TLM mobility, μ^TLM^, can thus be written as

4 $$\mu^{\mathrm{TLM}}=\frac{\frac{1}{R_{sh}}}{n\left(V_{\mathrm{GSO}}\right)}$$

It should be noted that the Drude model is not directly applicable for small *V*_GSO_ ^47^ and is only applicable when *n ≫ n*_0_, where *n*_0_ is the residual charge-carrier concentration at minimum conductivity (Dirac point). The hole and electron mobilities are extracted at carrier concentrations of −1 × 10^12^ cm^–2^ and 1 × 10^12^ cm^–2^, respectively.

The mobility of the graphene channel can also be directly calculated using the Drude model on individual channels, where the sheet conductance can be estimated using the dimensions of the graphene channel:^48^

5 $$\mu^{\mathrm{Drude}}=\frac{1}{R_{sh}}\frac{1}{n\left(V_{\mathrm{GSO}}\right)}=\frac{L}{R_{\mathrm{channel}}W}\frac{1}{n\left(V_{\mathrm{GSO}}\right)}\approx \frac{I_{\mathrm{ds}}L}{V_{\mathrm{ds}}W}\frac{1}{n\left(V_{\mathrm{GSO}}\right)}$$

where *R*_sh_ = *R*_channel_ *W*/*L* and $R_{\mathrm{channel}}\approx \frac{V_{\mathrm{ds}}}{I_{\mathrm{ds}}}$ . This method neglects the contribution of *R*_c_ and n_0_ and is only applicable when *R*_c_ ≪ *R*_channel_ and *n ≫ n*_0_.

Alternatively, the DTM can be used to estimate mobility.^26^ In this case, mobility is extracted from the gate-voltage-dependent transconductance by using the following equation:

6 $$\mu^{\mathrm{DTM}}=g_{m}\frac{L}{WV_{\mathrm{ds}}C_{g}\left(V_{\mathrm{GSO}}\right)}$$

where *g*_m_ = ∂*I*_ds_/∂*V*_GSO_ is transconductance. This method also neglects the contribution of *R*_c_ and is only applicable when *R*_c_ ≪ *R*_channel_. The DTM is applied to individual GFET transfer curves. Typically, hole and electron mobilities are taken from the maxima of the hole and electron conduction branches, respectively. Here we define the hole μ^DTM^ at a carrier concentration of −1 × 10^12^ cm^–2^ to be comparable with the other methods.

Equation 2 can be modified to consider the total charge-carrier concentration, defined as $n_{\mathrm{tot}}=\sqrt{n_{0}^{2}+n^{2}}$ , where *n*_0_ is ideally zero for a disorder-free graphene.^37^ Using this definition and eq 3, we can directly model *R*_total_ from eq 1 (see Supplementary Information for more details):

7 $$R_{\mathrm{total}}\left(V_{\mathrm{GSO}}\right)=2R_{c}+\frac{L}{W}\frac{1}{\mu^{\mathrm{FTM}}e\sqrt{(n{\left(V_{\mathrm{GSO}}\right)}^{2}+n_{0}^{2}}}$$

Equation 7 is a commonly used model for mobility and contact resistance extraction.^37^ Here, we have an additional factor of 2 with the *R*_*c*_ term to be consistent with eq 1. Contact resistance, mobility, and residual carrier concentration can be extracted by fitting eq 7 against measured transfer curves. We define μ^FTM^ to differentiate between the result of directly applying eq 5. See Supplementary Information Section 3 for more details.

Finally, we also compare the mobility of the graphene measured using the Hall effect on square van der Pauw test structures by using Ecopia HMS-500.

Transfer curve hysteresis has been measured by sweeping the gate voltage back and forth. The difference between the Dirac peak voltages, normalized by dividing with the total gate-voltage range, is used as the hysteresis value according to the following equation:

8 $$Hysteresis=\frac{\left(V_{Dirac,f}-V_{Dirac,b}\right)}{V_{GS,range}}$$

gCBK structures have been utilized as an alternative approach to measure contact resistance directly to compare with the values obtained by using the gated TLM. The measurement principle is presented in Supplementary Information Section 4. A current is applied between contacts 1 and 2, while the voltage drop across the contact is measured between contacts 3 and 4. Contact resistance can then be obtained according to the following equation:

9 $$R_{c}=\frac{V_{34}}{I}$$

where *V*_34_ is the voltage drop between contacts 3 and 4 and *I* is the current between points 1 and 2. Here, we normalize the contact resistance for the graphene devices by the width of the graphene instead of the contact area as typically done with gCBK structures.^36^ *R*_c_ extracted using the other methods is also normalized in this manner to define specific contact resistance:

10 $$\rho_{c}=R_{c}W$$

Contact resistance values extracted from gated TLM (contract front resistance) and gCBK (interfacial contact resistance) should be seen as complementary to each other, as shown by König et al.^33^ when using a modified CBK structure to measure contact interface resistance. By using this specific area contact resistivity *ρ*_c,A_, transfer length *L*_T_ and graphene sheet resistance on the contact metal *R*_sk_ can be extracted.

Finally, the yield of the devices has been defined with on/off ratio, maximum channel resistance, and gate leakage current parameters. On/off ratio should be higher than 5, *R*_sh_ = *R*_channel_ *W*/*L* smaller than 10 kΩ at *V*_Dirac_, and gate leakage current smaller than 10 nA for the device to be accepted as a working device.

### AFM and SEM Characterization

Atomic force microscopy (AFM) and scanning electron microscopy (SEM) were used to analyze the topographical results of the process optimization efforts to reduce both the contact and back-gate roughness, step heights, and edge discontinuities. Here, edge discontinuity refers to additional material at the edges of metal features. SEM was used to qualitatively analyze layer step heights and edge discontinuities, and AFM was used to quantitatively measure surface roughness, layer step heights, and edge discontinuities. In addition, AFM was used to analyze the amount of resist residue on the graphene channel by measuring the total thickness of the channel after the graphene patterning step. SEM was also utilized to measure the actual channel dimensions after device fabrication to improve the accuracy of the analysis.

## Supplementary Material



## Notes

GFET fabrication process flow, capacitance measurements, comparison of different GFET models, contact resistance from cross-bridge Kelvin structures, and comparison of contact resistance extraction methods (PDF)

## References

[1] Novoselov K. S., Geim A. K., Morozov S. V., Jiang D., Zhang Y., Dubonos S. V., Grigorieva I. V., Firsov A. A. (2004). Electric Field Effect
in Atomically Thin Carbon Films. Science.

[2] Li S., Wang Z., Robertz B., Neumaier D., Txoperena O., Maestre A., Zurutuza A., Bower C., Rushton A., Liu Y., Harris C., Bessonov A., Malik S., Allen M., Medina-Salazar I., Ryhänen T., Lemme M. C. (2025). Graphene-PbS Quantum
Dot Hybrid Photodetectors from 200 Mm Wafer Scale Processing. Sci. Rep..

[3] Cabral P. D., Domingues T., Machado G., Chicharo A., Cerqueira F., Fernandes E., Athayde E., Alpuim P., Borme J. (2020). Clean-Room
Lithographical Processes for the Fabrication of Graphene Biosensors. Materials.

[4] Soikkeli M., Murros A., Rantala A., Txoperena O., Kilpi O.-P., Kainlauri M., Sovanto K., Maestre A., Centeno A., Tukkiniemi K., Gomes Martins D., Zurutuza A., Arpiainen S., Prunnila M. (2023). Wafer-Scale Graphene
Field-Effect Transistor Biosensor Arrays with Monolithic CMOS Readout. ACS Appl. Electron. Mater..

[5] Vieira N. C. S., Borme J., Machado G., Cerqueira F., Freitas P. P., Zucolotto V., Peres N. M. R., Alpuim P. (2016). Graphene Field-Effect
Transistor Array with Integrated Electrolytic Gates Scaled to 200
Mm. J. Phys.: Condens. Matter.

[6] Rahimi S., Tao L., Chowdhury S., Park F., Jouvray S., Buttress A., Rupesinghe S., Teo N., Akinwande K. D. (2014). Toward
300 Mm Wafer-Scalable High-Performance Polycrystalline Chemical Vapor
Deposited Graphene Transistors. ACS Nano.

[7] Wu C., Brems S., Yudistira D., Cott D., Milenin A., Vandersmissen K., Maestre A., Centeno A., Zurutuza A., Van Campenhout J. (2023). Wafer-Scale Integration of Single Layer Graphene
Electro-Absorption Modulators in a 300 Mm CMOS Pilot Line. Laser Photon. Rev..

[8] Lisker M., Lukosius M., Fraschke M., Kitzmann J., Dabrowski J., Fursenko O., Kulse P., Schulz K., Krüger A., Drews J., Schulze S., Wolansky D., Schubert A. M., Katzer J., Stolarek D., Costina I., Wolff A., Dziallas G., Coccetti F., Mai A. (2019). Processing and Integration
of Graphene in a 200 mm Wafer Si Technology Environment. Microelectron. Eng..

[9] Canto B., Otto M., Maestre A., Centeno A., Zurutuza A., Robertz B., Reato E., Chmielak B., Stoll S. L., Hemmetter A., Schlachter F., Ehlert L., Li S., Neumaier D., Rinke G., Wang Z., Lemme M. C. (2025). Multi-Project
Wafer Runs for Electronic Graphene Devices in the European 2D-Experimental
Pilot Line Project. Nat. Commun..

[10] Neumaier D., Pindl S., Lemme M. C. (2019). Integrating
Graphene into Semiconductor
Fabrication Lines. Nat. Mater..

[11] Goossens S., Navickaite G., Monasterio C., Gupta S., Piqueras J. J., Pérez R., Burwell G., Nikitskiy I., Lasanta T., Galán T., Puma E., Centeno A., Pesquera A., Zurutuza A., Konstantatos G., Koppens F. (2017). Broadband Image Sensor Array Based
on Graphene–CMOS
Integration. Nat. Photonics.

[12] Mortazavi
Zanjani S. M., Holt M., Sadeghi M. M., Rahimi S., Akinwande D. (2017). 3D Integrated Monolayer Graphene–Si CMOS RF
Gas Sensor Platform. Npj 2D Mater. Appl..

[13] Dockx K., Barnes M. D., Wehenkel D. J., van Rijn R., van der
Zant H. S. J., Buscema M. (2024). Strong Doping Reduction on Wafer-Scale
CVD Graphene Devices via Al _2_ O _3_ ALD Encapsulation. Nanotechnology.

[14] Liu, Y. ; Chang, J. ; Lin, L. A Flexible Graphene FET Gas Sensor Using Polymer as Gate Dielectrics. In Proceedings of the IEEE International Conference on Micro Electro Mechanical Systems (MEMS); IEEE, 2014; pp. 230–233. DOI: 10.1109/MEMSYS.2014.6765617.

[15] Soikkeli M., Kurppa K., Kainlauri M., Arpiainen S., Paananen A., Gunnarsson D., Joensuu J. J., Laaksonen P., Prunnila M., Linder M. B., Ahopelto J. (2016). Graphene Biosensor
Programming with Genetically Engineered Fusion Protein Monolayers. ACS Appl. Mater. Interfaces.

[16] Lee S.-J., Cho W.-J. (2025). Emerging Dual-Gate
FET Sensor Paradigm for Ultra-Low
Concentration Cortisol Detection in Complex Bioenvironments. Biosensors.

[17] Ahn J.-H., Choi B., Choi S.-J. (2020). Understanding the Signal Amplification
in Dual-Gate FET-Based Biosensors. J. Appl.
Phys..

[18] Smith A. D., Wagner S., Kataria S., Malm B. G., Lemme M. C., Ostling M. (2017). Wafer-Scale Statistical
Analysis of Graphene Field-Effect
Transistors—Part II: Analysis of Device Properties. IEEE Trans. Electron. Devices.

[19] Smith A. D., Wagner S., Kataria S., Malm B. G., Lemme M. C., Ostling M. (2017). Wafer-Scale Statistical
Analysis of Graphene FETs—Part
I: Wafer-Scale Fabrication and Yield Analysis. IEEE Trans. Electron. Devices.

[20] Kong Q., Wang X., Xia L., Wu C., Feng Z., Wang M., Zhao J. (2017). Achieving Low Contact
Resistance
by Engineering a Metal–Graphene Interface Simply with Optical
Lithography. ACS Appl. Mater. Interfaces.

[21] Lisker M., Lukosius M., Kitzmann J., Fraschke M., Wolansky D., Schulze S., Lupina G., Mai A. (2018). Contacting Graphene
in a 200 mm Wafer Silicon Technology Environment. Solid State Electron..

[22] Elviretti M., Lisker M., Lukose R., Lukosius M., Akhtar F., Mai A. (2022). Reliable Metal–Graphene Contact
Formation Process Flows in
a CMOS-Compatible Environment. Nanoscale Adv..

[23] Kim Y. J., Lee Y. G., Jung U., Lee S., Lee S. K., Lee B. H. (2015). A Facile Process to Achieve Hysteresis-Free
and Fully
Stabilized Graphene Field-Effect Transistors. Nanoscale.

[24] Tien D. H., Park J.-Y., Kim K. B., Lee N., Seo Y. (2016). Characterization
of Graphene-Based FET Fabricated Using a Shadow Mask. Sci. Rep..

[25] Gahoi A., Kataria S., Driussi F., Venica S., Pandey H., Esseni D., Selmi L., Lemme M. C. (2020). Dependable Contact
Related Parameter Extraction in Graphene–Metal Junctions. Adv. Electron. Mater..

[26] Zhong H., Zhang Z., Xu H., Qiu C., Peng L.-M. (2015). Comparison
of Mobility Extraction Methods Based on Field-Effect Measurements
for Graphene. AIP Adv..

[27] Capista, D. ; Lukose, R. D. ; Majnoon, F. ; Lisker, M. ; Wenger, C. ; Lukosius, M. Optimization of the Metal Deposition Process for the Accurate Estimation of Low Metal-Graphene Contact-Resistance. In 2024 47th MIPRO ICT and Electronics Convention (MIPRO); IEEE, 2024; 1561–1565. DOI: 10.1109/MIPRO60963.2024.10569895.

[28] Miseikis V., Bianco F., David J., Gemmi M., Pellegrini V., Romagnoli M., Coletti C. (2017). Deterministic Patterned Growth of
High-Mobility Large-Crystal Graphene: A Path towards Wafer Scale Integration. 2D Mater..

[29] Mackenzie D. M. A., Buron J. D., Whelan P. R., Caridad J. M., Bjergfelt M., Luo B., Shivayogimath A., Smitshuysen A. L., Thomsen J. D., Booth T. J., Gammelgaard L., Zultak J., Jessen B. S., Bøggild P., Petersen D. H. (2017). Quality Assessment of Graphene: Continuity, Uniformity,
and Accuracy of Mobility Measurements. Nano
Res..

[30] Qing F., Shu Y., Qing L., Niu Y., Guo H., Zhang S., Liu C., Shen C., Zhang W., Mao S. S., Zhu W., Li X. (2018). A General and Simple
Method for Evaluating the Electrical Transport
Performance of Graphene by the van Der Pauw–Hall Measurement. Sci. Bull..

[31] Nagashio K., Nishimura T., Kita K., Toriumi A. (2010). Contact Resistivity
and Current Flow Path at Metal/Graphene Contact. Appl. Phys. Lett..

[32] Wang S., Mao D., Jin Z., Peng S., Zhang D., Shi J., Wang X. (2015). A More Reliable Measurement Method for Metal/Graphene Contact Resistance. Nanotechnology.

[33] Konig M., Ruhl G., Gahoi A., Wittmann S., Preis T., Batke J.-M., Costina I., Lemme M. C. (2019). Accurate Graphene-Metal
Junction Characterization. IEEE J. Electron
Devices Soc..

[34] Wang S., Mao D., Muhammad A., Peng S., Zhang D., Shi J., Jin Z. (2016). Characterization of the Quality of Metal–Graphene Contact
with Contact End Resistance Measurement. Appl.
Phys. A.

[35] Zhang Z., Xu H., Zhong H., Peng L.-M. (2012). Direct Extraction of Carrier Mobility
in Graphene Field-Effect Transistor Using Current-Voltage and Capacitance-Voltage
Measurements. Appl. Phys. Lett..

[36] Cha J., Son J., Hong J. (2022). A Bottom-Electrode
Contact: The Most Suitable Structure
for Graphene Electronics. Adv. Mater. Interfaces.

[37] Kim S., Nah J., Jo I., Shahrjerdi D., Colombo L., Yao Z., Tutuc E., Banerjee S. K. (2009). Realization of a High Mobility Dual-Gated
Graphene Field-Effect Transistor with Al2O3 Dielectric. Appl. Phys. Lett..

[38] Jeppson K., Asad M., Stake J. (2021). Mobility Degradation and Series Resistance
in Graphene Field-Effect Transistors. IEEE Trans.
Electron. Devices.

[39] Xia F., Perebeinos V., Lin Y., Wu Y., Avouris P. (2011). The Origins
and Limits of Metal–Graphene Junction Resistance. Nat. Nanotechnol..

[40] Venica S., Driussi F., Gahoi A., Palestri P., Lemme M. C., Selmi L. (2018). On the Adequacy of
the Transmission Line Model to Describe the Graphene–Metal
Contact Resistance. IEEE Trans. Electron. Devices.

[41] Nouchi R., Saito T., Tanigaki K. (2012). Observation of Negative Contact Resistances
in Graphene Field-Effect Transistors. J. Appl.
Phys..

[42] Huang T.-J., Spencer A., Ingraham L., Pacheco-Sanchez A., Puchades I. (2026). Raised or Recessed? Finding the Optimal
Gate Architecture
for Improving the Static Performance of Graphene Transistors. Nanoscale Adv..

[43] Yu H., Schaekers M., Schram T., Collaert N., De Meyer K., Horiguchi N., Thean A., Barla K. (2014). A Simplified Method
for (Circular) Transmission Line Model Simulation and Ultralow Contact
Resistivity Extraction. IEEE Electron. Device
Lett..

[44] Harrysson
Rodrigues I., Rorsman N., Vorobiev A. (2022). Mobility and Quasi-Ballistic
Charge Carrier Transport in Graphene Field-Effect Transistors. J. Appl. Phys..

[45] Silvestri A., Zayas-Arrabal J., Vera-Hidalgo M., Di Silvio D., Wetzl C., Martinez-Moro M., Zurutuza A., Torres E., Centeno A., Maestre A., Gómez J. M., Arrastua M., Elicegui M., Ontoso N., Prato M., Coluzza I., Criado A. (2023). Ultrasensitive Detection of SARS-CoV-2
Spike Protein by Graphene Field-Effect Transistors. Nanoscale.

[46] Gao Z., Kang H., Naylor C. H., Streller F., Ducos P., Serrano M. D., Ping J., Zauberman J., Rajesh, Carpick R. W. (2016). Scalable Production
of Sensor Arrays Based on High-Mobility Hybrid
Graphene Field Effect Transistors. ACS Appl.
Mater. Interfaces.

[47] Gosling J. H., Makarovsky O., Wang F., Cottam N. D., Greenaway M. T., Patanè A., Wildman R. D., Tuck C. J., Turyanska L., Fromhold T. M. (2021). Universal Mobility Characteristics of Graphene Originating
from Charge Scattering by Ionised Impurities. Commun. Phys..

[48] Brown M. A., Crosser M. S., Leyden M. R., Qi Y., Minot E. D. (2016). Measurement
of High Carrier Mobility in Graphene in an Aqueous Electrolyte Environment. Appl. Phys. Lett..

