# Supplementary material for: Graphene Field-Effect Transistor Process and Analysis Optimization in 200 mm Pilot-Line Environment
Source: ACS Nano. 2026 Sep 9;20(37):25275–89. doi: 10.1021/acsnano.6c03282 (PMC13602909; doi:10.1021/acsnano.6c03282)
Supplement: Supplementary file 1 [file nn6c03282_si_001.pdf]

# Supplementary

# Graphene Field-Effect Transistor Process and Analysis Optimization in 200 mm Pilot Line Environment

*Anton Murros[1§], Miika Soikkeli[1*§], Anni Virta[1], Arantxa Maestre[2,3], Leire Morillo[2], Alba Centeno[2], Amaia Zurutuza[2], Olli-Pekka Kilpi[1].*

[1]VTT Technical Research Centre of Finland Ltd, P.O. Box 1000, FI-02044 VTT, Espoo, Finland

[2]Graphenea S.A., Paseo Mikeletegi 83, 20009-San Sebastian, Spain

[3]ASM International, Kapeldreef 75, 3001 Leuven, Belgium

[§]These authors contributed equally to this work.

KEYWORDS: graphene, field-effect transistor, wafer-scale, processing, analysis, mobility, contact resistance

**Corresponding Author**

* E-mail: miika.soikkeli@vtt.fi

# Process flow

Graphene FET fabrication process flow consists of bottom gate, bottom contact, CVD graphene, encapsulation and channel opening process steps (Figure S1). Si wafers with different substrate dielectrics have been used as the starting wafers.

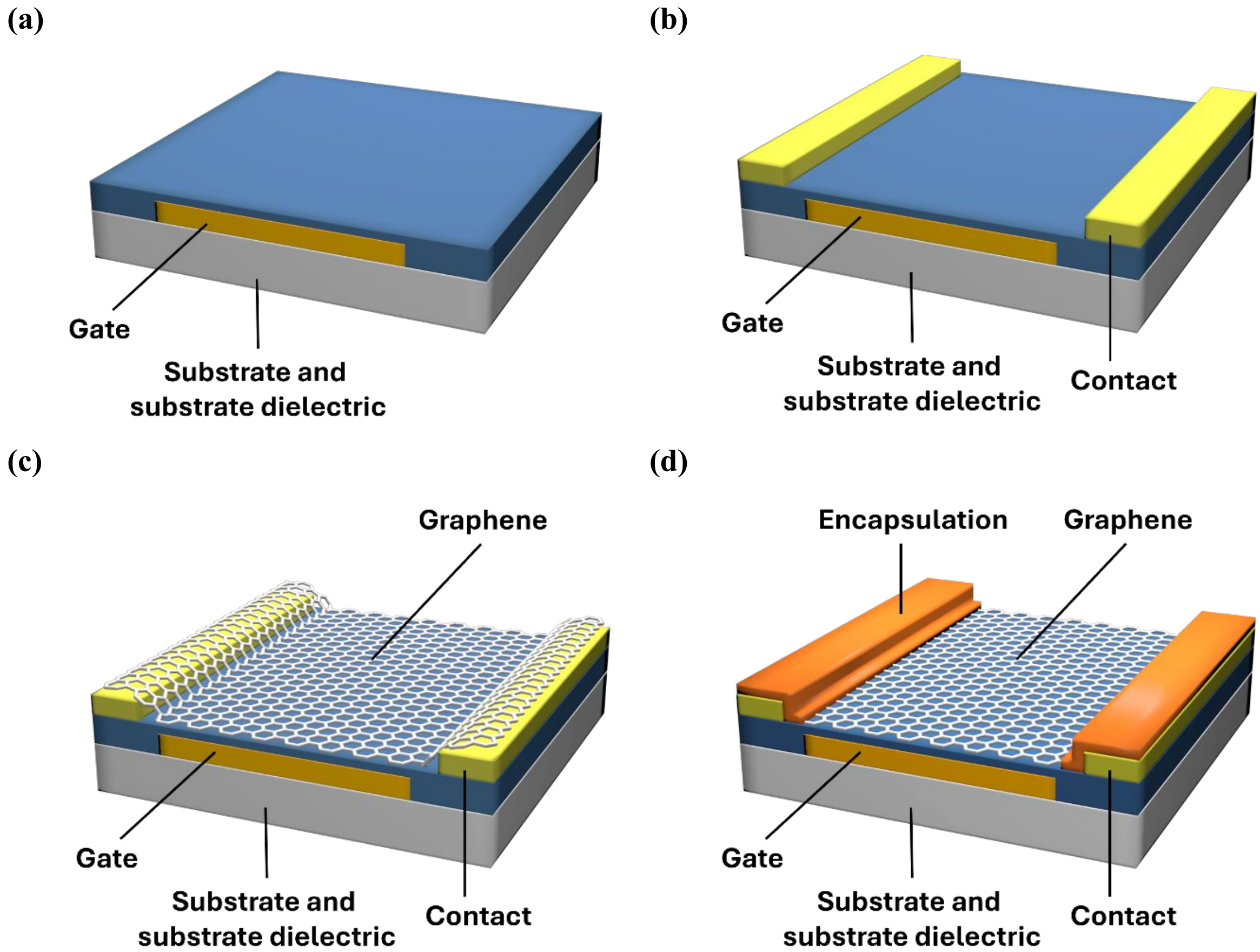


**Supplementary figure S1.** GFET fabrication process flow with a) local back gate, b) bottom contact, c) CVD graphene and d) encapsulation and channel opening process steps.

# Capacitance measurement

Capacitance-voltage (CV) measurements were done to improve the mobility and carrier concentration extraction accuracy. The measurements were conducted for each wafer run and the corresponding capacitance per unit area and carrier concentration values were used in the calculations. Figure S2 shows the CV measurement result from Run 6 as an example. The measurements were performed with a B1500A Keysight Semiconductor Device Parameter Analyzer with an AC amplitude of 0.1 V. Figure S2a shows the gate capacitance per unit area for 10 kHz frequency as a function of normalized bias voltage $V_{GSO}$ measured between the gate and source electrodes. Normalized doping concentration (Figure S2b) can be calculated by using the measured capacitance values according to the following equation:

$$n(V_{GSO}) = \frac{1}{e} \int_0^{V_{GSO}} C_g(V)\ dV. \tag{S1}$$

**(a)** **(b)**

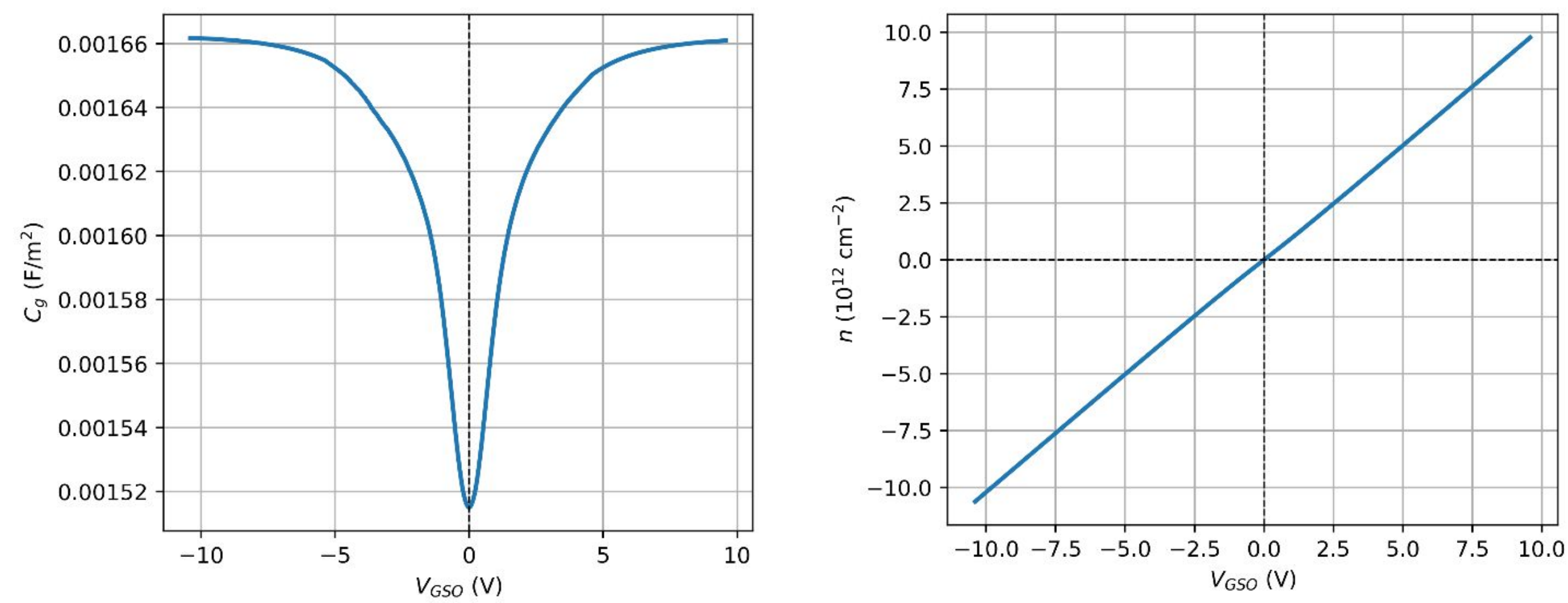


**Supplementary figure S2.** CV measurement results from Run 6 showing a) area normalized gate capacitance against $V_{GSO}$ measured at 10 kHz frequency and b) doping concentration against $V_{GSO}$.

# GFET models and comparison

### Mobility extraction equations

The MOSFET current in the triode region is typically defined as:

$$I_{ds} = \mu_n C_g \frac{W}{L}\left((V_{GS} - V_T)V_{ds} - \frac{V_{ds}^2}{2}\right). \tag{S1}$$

For GFETs we translate threshold voltage, $V_T$, into the Dirac peak voltage $V_{Dirac}$. We define gate voltage overdrive $V_{GSO} = V_{GS} - V_{Dirac}$. The $-V_{ds}^2/2$ term can be neglected as it is small when $V_{GSO} \neq 0$. Rearranging Equation (S1) we can define:

$$\mu_n = \frac{I_{ds}L}{V_{ds}W}\frac{1}{V_{GSO}C_g} = \frac{1}{R_{sh}}\frac{1}{V_{GSO}C_g} = \frac{\frac{1}{R_{sh}}}{V_{GSO}}\frac{1}{C_g}. \tag{S2}$$

where $V_{ds}/I_{ds} \approx R_{channel}$ and $R_{channel}W/L = R_{sh}$. Further defining carrier concentration $n = C_g V_{GSO}/e$, Equation (S2) has the same form as the base Drude model:

$$\frac{1}{R_{sh}} = \sigma_{sh} = \mu_n e n. \tag{S3}$$

The direct transconductance method (DTM) can be used to estimate mobility using the following equation:

$$\mu^{DTM} = g_m \frac{L}{W V_{ds} C_g}, \tag{S4}$$

where $g_m = \partial I_{ds}/\partial V_{GSO}$ is the transconductance. Assuming L, W and $V_{ds}$ are constant, we can rearrange the terms in Equation (S4) to show that the DTM method and Drude method define mobility in a similar way:

$$\mu^{DTM} = \frac{\partial\left(\frac{I_{ds}}{V_{ds}}\frac{L}{W}\right)}{\partial V_{GSO}}\frac{1}{C_g} = \frac{\partial\left(\frac{1}{R_{channel}}\frac{L}{W}\right)}{\partial V_{GSO}}\frac{1}{C_g} = \frac{\partial\left(\frac{1}{R_{sh}}\right)}{\partial V_{GSO}}\frac{1}{C_g}. \tag{S5}$$

We note that Equation (S5) is of the same form as Equation (S2) but without the differential operator. Returning to Equation (S3) and differentiating in terms of $V_{GSO}$ we obtain:

$$\frac{\partial\left(\frac{1}{R_{sh}}\right)}{\partial V_{GSO}} = \frac{\partial\left(\mu_n C_g V_{GSO}\right)}{\partial V_{GSO}} = \frac{\partial \mu_n}{\partial V_{GSO}} C_g V_{GSO} + \frac{\partial C_g}{\partial V_{GSO}} \mu_n V_{GSO} + \mu_n C_g. \tag{S6}$$

If $\mu_n$ and $C_g$ are assumed to be constant with respect to $V_{GSO}$, Equation (S6) simplifies to the same form as Equation (S5). We find that the transconductance Equation (S4) thereby neglects the contributions of other terms that vary with respect to $V_{GSO}$, which likely causes the difference in extracted mobility values. Note that the carrier concentration or cate capacitance can be replaced in these formulas with the measured values as done in the main text.

For the FTM equation de the total charge carrier concentration can be written as:

$$n_{tot} = \sqrt{{n_0}^2 + n^2}\,, \tag{S7}$$

where $n_0$ is residual charge carrier concentration and n is the gate inducted charge carrier concentration. The effect of including the $n_0$ term is to stabilize the Drude equation near the Dirac peak.

n is the gate induced charge carrier or doping concentration, which can be formulated with measured gate capacitance per unit area $C_g$:

$$n(V_{GSO}) \;=\; \frac{1}{e}\int_0^{V_{GSO}} C_g(V)\; dV. \tag{S8}$$

The equations S7 and S8 can be utilized together with the Drude formula to obtain the following equation:

$$\sigma = e\mu^{FTM}\sqrt{n_0^2 + n^2}, \tag{S9}$$

where $\mu^{FTM}$ is carrier mobility. This equation can be further converted to sheet resistance:

$$R_{sh} = \frac{L}{W}\frac{1}{e\mu^{FTM}\sqrt{n_0^2+n^2}}. \tag{10}$$

By adding the contact resistance and substituting the equation S8 the total resistance of the graphene channel can be written as:

$$R_{total}(V_{GSO}) = 2R_c + \frac{L}{W}\frac{1}{\mu^{FTM}e\sqrt{(\mathrm{n}(V_{GSO})^2 + n_0^2}}. \tag{S11}$$

where $R_c$ is the contact resistance. Here we have an additional factor of 2 with the $R_c$ term to be consistent with our other equations.

## The impact of device dimensions

Graphene channel dimensions have a larger effect on the single GFET methods (FTM, DTM, Drude method), than the gated TLM method. The linear fitting process of the gated TLM method removes the effect of channel length dependency as shown for the gated TLM method with Drude (Figure S3a) and transconductance formulas (Figure S3b). The single channel methods FTM (Figure S3c), Drude (Figure S3e) and DTM (Figure S3g) show also quite uniform mobility values with respect to the channel width. The effect of graphene channel length is greater in smaller dimensions, which is shown for FTM (Figure S3d), Drude (Figure S3f) and DTM (Figure S3h).

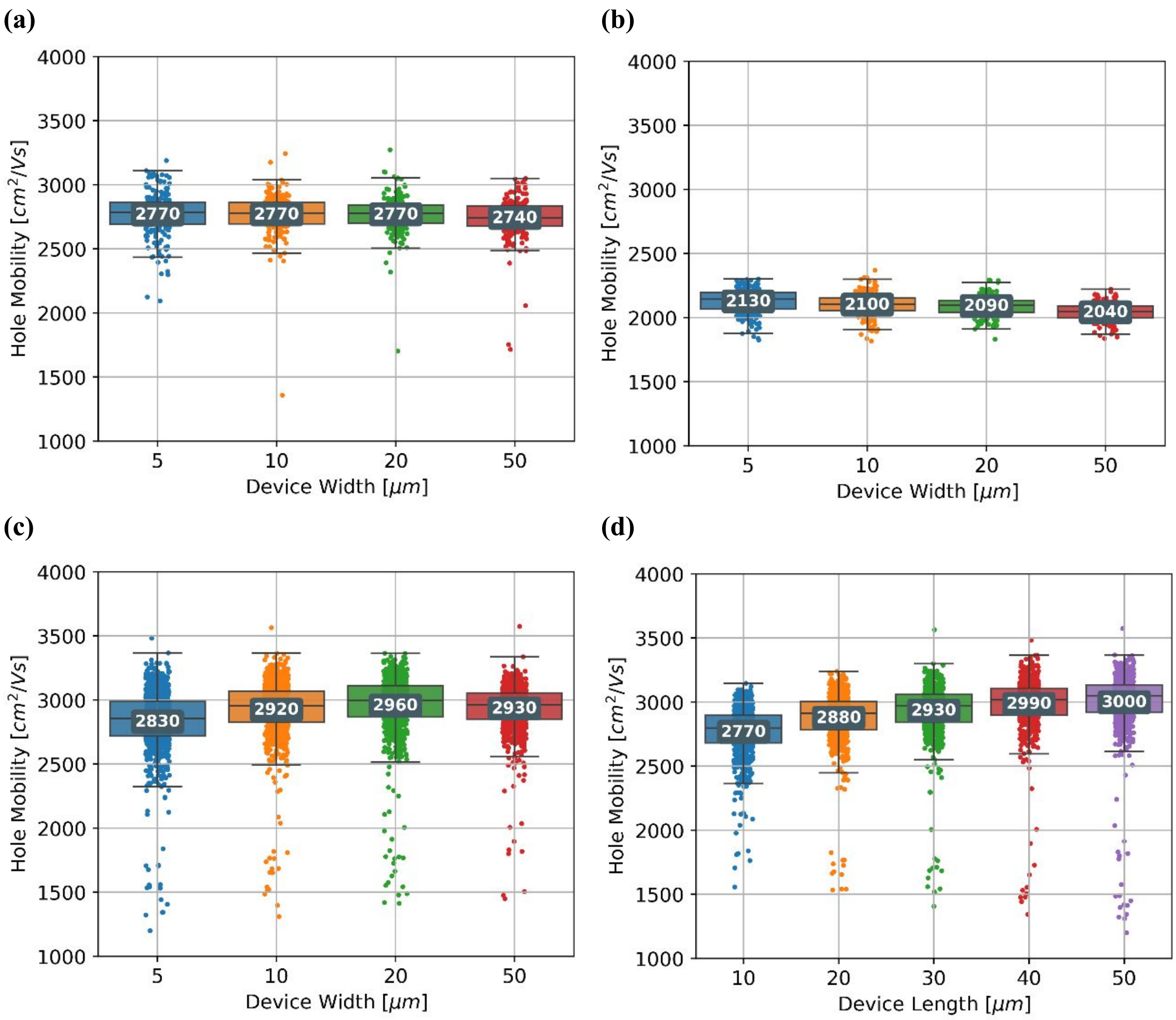

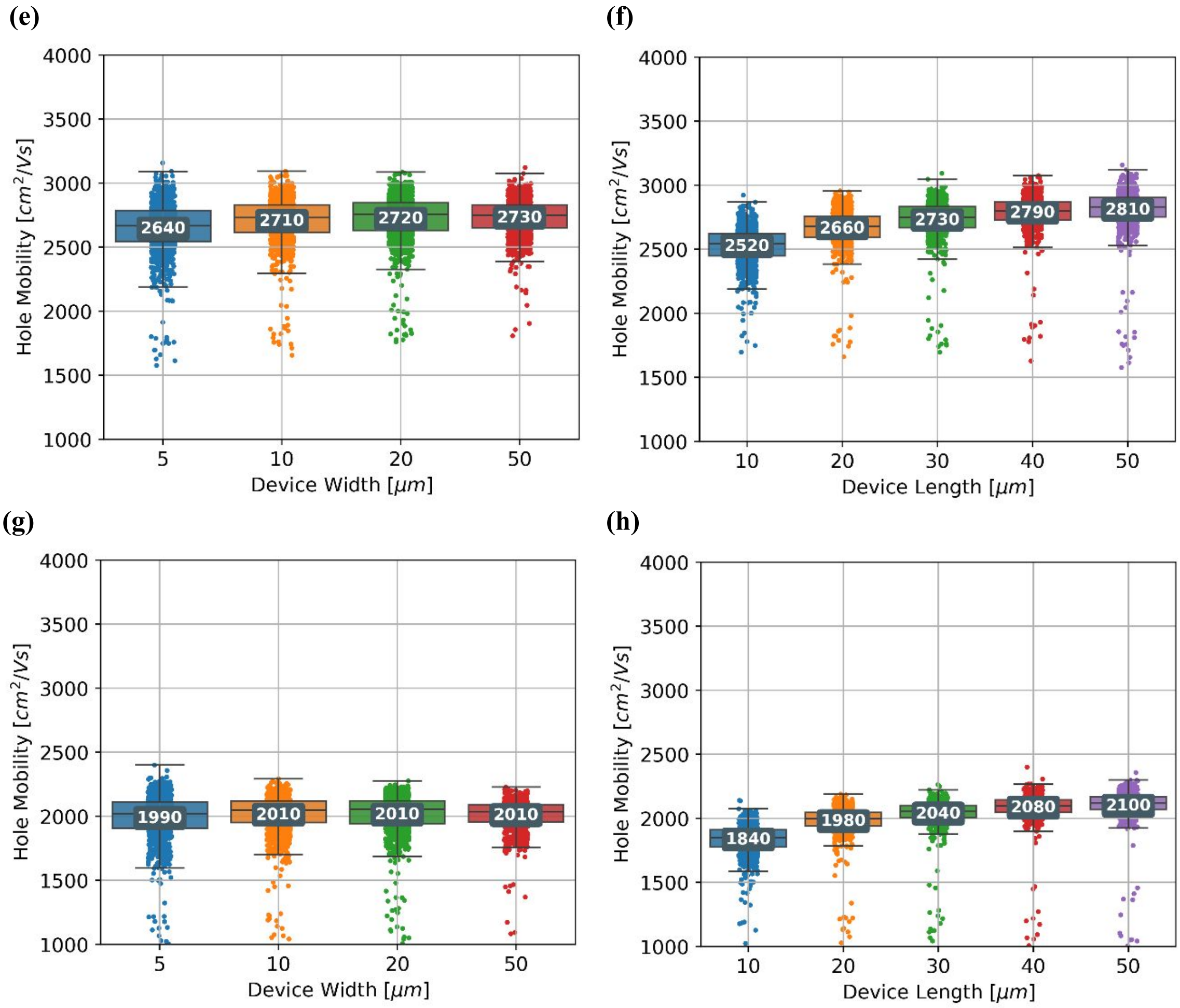


**Supplementary figure S3.** Extracted hole mobility statistics against graphene channel dimensions. a) The impact of device width with the gated TLM method using Drude formula. b) The impact of device width with gated TLM method using transconductance formula. c) The impact of device width using FTM. d) The impact of device length using FTM. e) The impact of device width using Drude. f) The impact of device length using Drude. g) The impact of device width using DTM. h) The impact of device length using DTM. Inset values are average values of each dataset.

For the DTM mobilities are typically defined using the maxima of the hole and electron conduction branches, which differs from the Drude method that extracts these values from fixed carrier concentrations. Figure S4 shows that there is no significant difference in the extracted hole mobility in the DTM using maxima or specific carrier concentrations with our data set taken at $-1 \times 10^{12}$ $cm^{-2}$ but it still should be considered when doing comparisons.

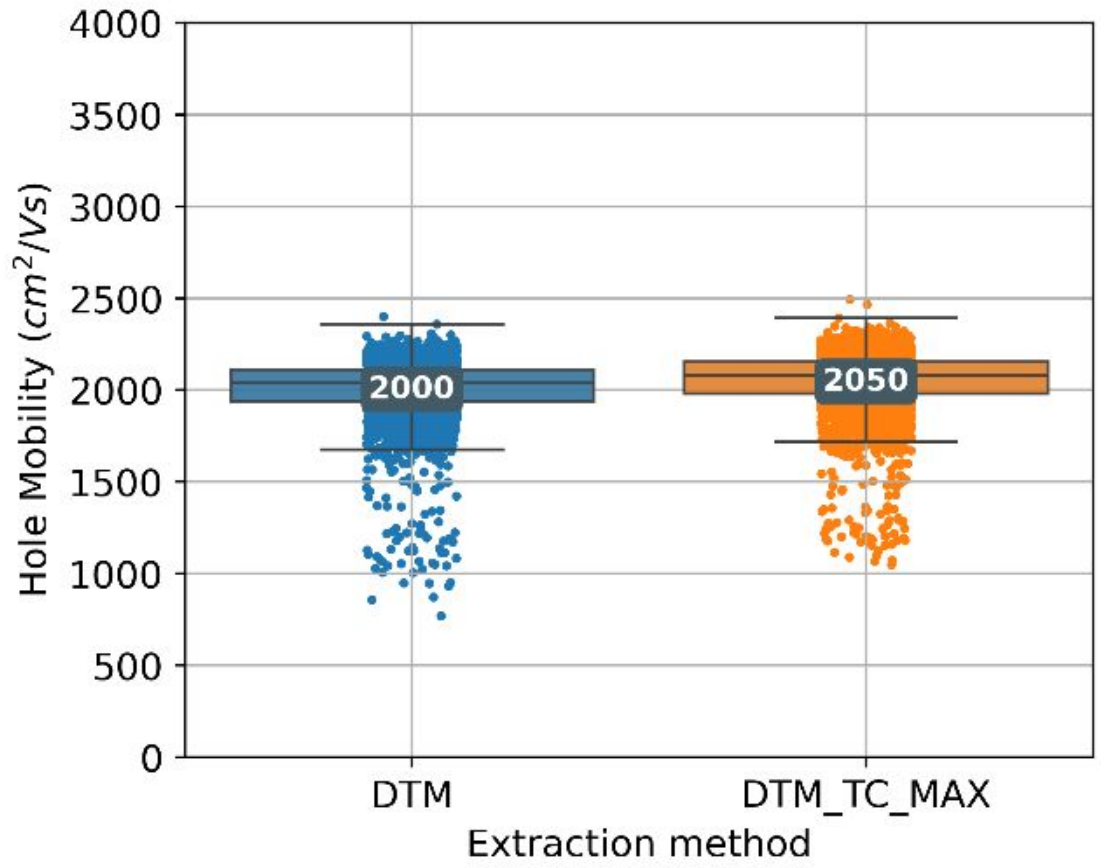


**Supplementary figure S4.** Extracted hole mobility using DTM, with either the mobilities being defined at the maxima of the hole conduction branch or from a carrier concentration of $-1 \times 10^{12}$ $cm^{-2}$ (-, doping). Inset values are average values of each dataset.

# Contact resistance from Cross-bridge Kelvin structures

CBK structures have been utilized as an alternative approach to measure contact resistance directly to compare with the values obtained by using the gated TLM method. The measurement principle is presented in Figure S5. A current is applied between contacts 1 and 2 while the voltage drop over the contact is measured between contacts 3 and 4. Contact resistance can then be obtained according to the following equation:

$$R_c = \frac{V_{34}}{I}, \tag{S10}$$

where $V_{34}$ is the voltage drop between contact 3 and 4 and $I$ is the current between points 1 and 2. The contact resistance for the graphene devices should be normalized by using the length of the contact perimeter instead of the contact area as typically done with CBK structures.[1] This can be done by using the following equation:

$$\rho_c = R_c W, \tag{S11}$$

where $W$ is the width of the contact.

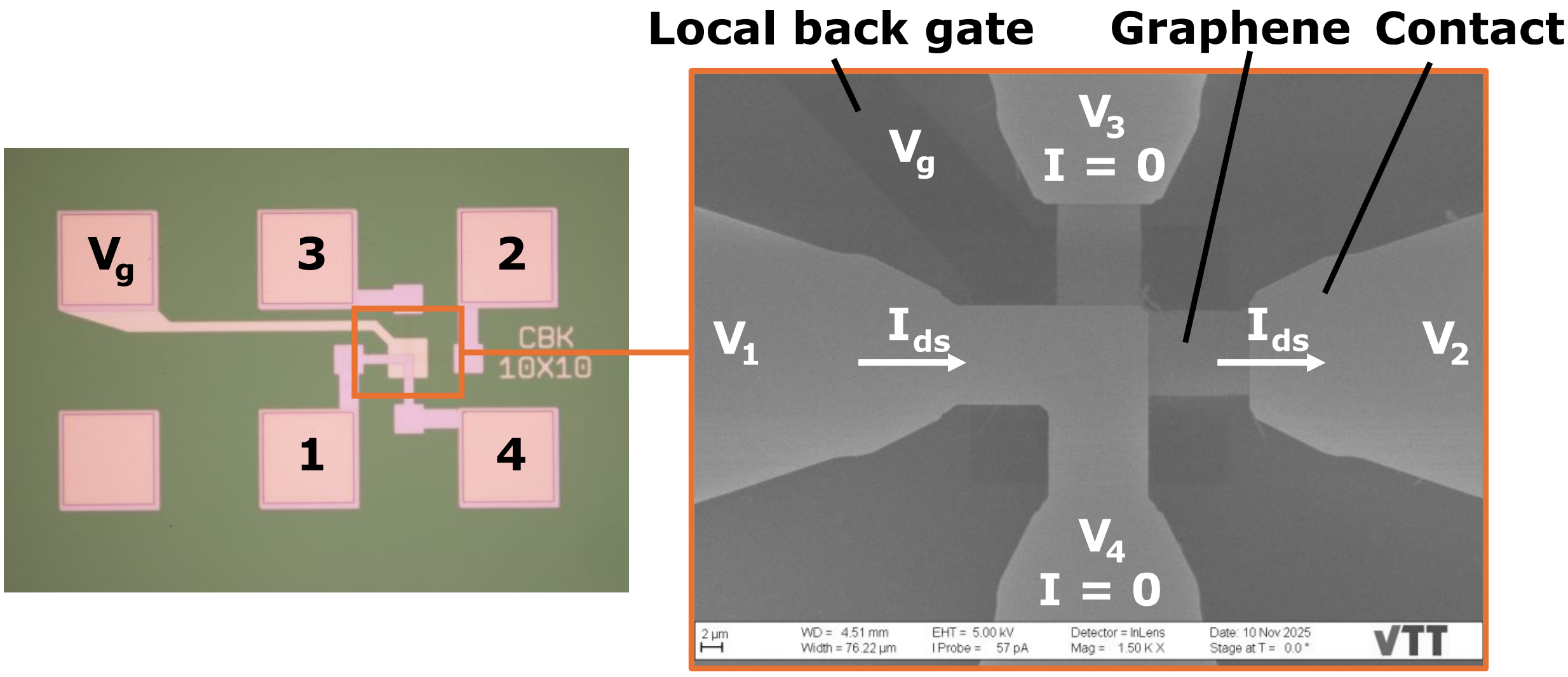


**Supplementary figure S5.** Contact resistance extraction principle from a CBK structure. A current is applied between contacts 1 and 2 while the voltage drop over the contact is measured between contacts 3 and 4.

## Comparison of contact resistance extraction methods

GFETs analysed with the gated TLM method and gCBK structures have been further studied by comparing the impact of device widths and carrier concentration on the extracted values. The values extracted from the GFETs with the gated TLM method (Figure S6a) show clear dependency on the channel width while the gCBK structures (Figure S6b) do not. When the same devices are analysed as a function of the higher hole and electron carrier concentration and around Dirac peak the values extracted from GFETs (Figure S7a) show clearly higher dependency on the carrier concentration when compared to the gCBK devices (Figure S7b).

**(a)** **(b)**

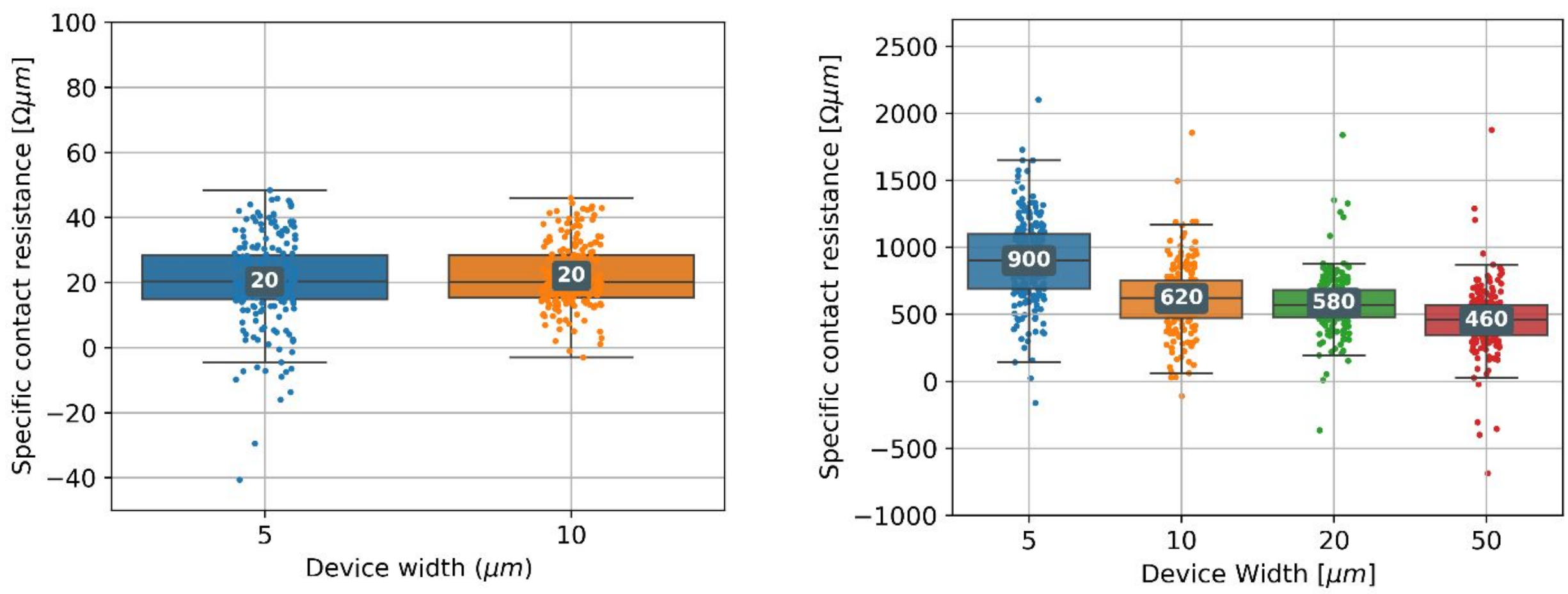


**Supplementary figure S6.** Contact resistance values as a function of the device width extracted from a) GFETs with gated TLM method and b) gCBK structures. Values extracted from GFETs show dependency to the device width while the values from gCBK structures do not.

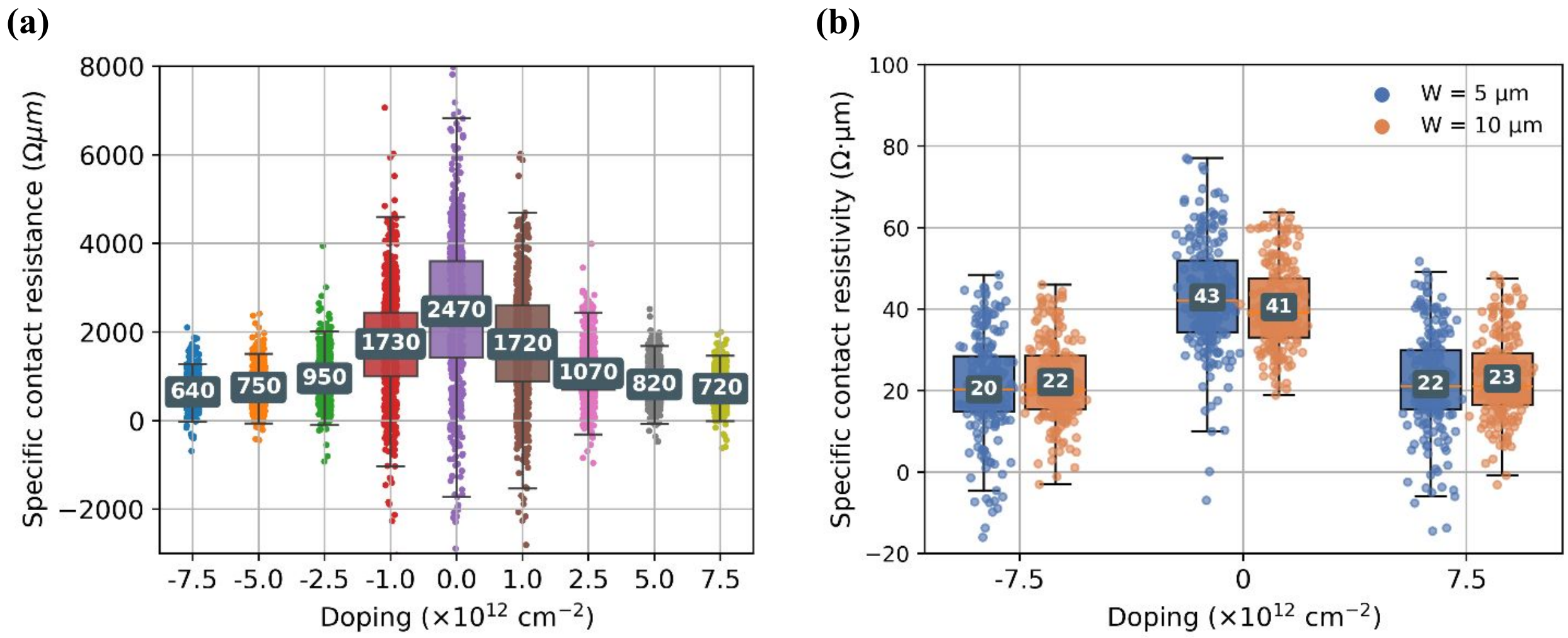


**Supplementary figure S7.** Contact resistance values as a function of the carrier density extracted from a) GFETs with gated TLM method and b) gCBK structures. Values extracted from GFETs show stronger dependency to the carrier concentration than the values from gCBK structures.

# REFERENCES


(1) Cha, J.; Son, J.; Hong, J. A Bottom-Electrode Contact: The Most Suitable Structure for Graphene Electronics. *Adv. Mater. Interfaces* **2022**, *9* (6). https://doi.org/10.1002/admi.202102207.
